# Probiotic bacteria-released extracellular vesicles enhance macrophage phagocytosis in polymicrobial sepsis by activating the FPR1/2 pathway

**DOI:** 10.1186/s10020-024-00959-9

**Published:** 2024-11-14

**Authors:** Ruiyao Zhu, Yu Zhang, Xiaohong Wang, Benjamin D. Liu, Debabrata Chowdhury, Zhixin Li, Mingliang Pan, Tianqing Peng, Jing Chen, Wei Huang, Liying Zhan, Guo-Chang Fan

**Affiliations:** 1 Department of Critical Care Medicine, Renmin Hospital of Wuhan University, Wuhan, Hubei China; 2 Department of Pharmacology and Systems Physiology, University of Cincinnati College of Medicine, 231 Albert Sabin Way, Cincinnati, OH 45267-0575 USA; 3 Department of Infection Prevention and Control, Renmin Hospital of Wuhan University, Wuhan, Hubei China; 4 Department of Chemistry and Biochemistry, The Ohio State University College of Arts and Sciences, Columbus, OH USA; 5 Centre for Critical Illness Research, Lawson Health Research Institute, London, ON Canada; 6 Division of Biomedical Informatics, Cincinnati Children’s Hospital Medical Center, Cincinnati, OH USA; 7 Division of Cardiovascular Health and Disease, Department of Internal Medicine, University of Cincinnati College of Medicine, Cincinnati, OH USA

**Keywords:** Sepsis, Macrophage phagocytosis, Probiotic *Lactobacillus rhamnosus* GG, Extracellular vesicles, FPR1/2 signaling

## Abstract

**Background:**

Sepsis-induced organ failure and high mortality are largely ascribed to the failure of bacterial clearance from the infected tissues. Recently, probiotic bacteria-released extracellular vesicles (BEVs) have been implicated as critical mediators of intercellular communication which are widely involved in the regulation of the inflammatory response. However, their functional role in macrophage phagocytosis during sepsis has never been explored.

**Methods:**

BEVs were collected from three different strains of probiotics including *Lactiplantibacillus plantarum* WCFS1 (LP WCFS1), *Lactobacillus rhamnosus* Gorbach-Goldin (LGG), and *Escherichia coli* Nissle 1917 (EcN), or from LGG cultured under three pH conditions (pH5-acid, pH6.5-standard, pH8-akaline) through differential centrifugation, filtration, and ultracentrifugation of their culture supernatants. In vitro phagocytosis was measured in Raw264.7 cells and bone marrow-derived macrophages using pHrodo red *E. coli* BioParticles. The in vivo therapeutic effects of BEVs were tested using a feces-injection-in-peritoneum (FIP) model of polymicrobial sepsis.

**Results:**

LGG-derived EVs (BEV^LGG^) were the best among these three probiotics BEVs in stimulating macrophages to take up bacteria. Furthermore, BEV^LGG^ collected from pH8 culture condition (BEV^pH8^) exhibited the strongest capacity of phagocytosis, compared with BEV^pH5^ and BEV^pH6.5^. Treatment of septic mice with BEV^pH8^ significantly prolonged animal survival; increased bacterial clearance from the blood, peritoneal lavage fluid, and multiple organs; and decreased serum levels of pro-inflammatory cytokines/chemokines, as well as reduced multiple organ injuries, in comparison with control-treated septic mice. Mechanistically, RNA-seq and bioinformatic analysis identified that the FPR1/2 signaling was remarkably activated, along with its downstream pathways (PI3K-Akt-MARCO and NADPH-ROS) in BEV^pH8^-treated macrophages, compared with control cells. Accordingly, pre-addition of Boc2, a specific antagonist of FPR1/FPR2, to macrophages significantly attenuated BEV^pH8^-mediated phagocytosis, compared to controls.

**Conclusions:**

This study demonstrates that LGG-derived BEVs may have therapeutic effects against sepsis-induced organ injury and mortality through enhancing FPR1/2-mediated macrophage phagocytosis.

**Supplementary Information:**

The online version contains supplementary material available at 10.1186/s10020-024-00959-9.

## Introduction

Sepsis is characterized by life-threatening organ dysfunction caused by a dysregulated host response to infection (Singer et al. 2016). The incidence of sepsis is on the rise worldwide due to population aging, the increased prevalence of chronic diseases, invasive medical procedures, and healthcare-associated infections (Font et al. 2020). Currently, sepsis management relies heavily on antibiotics, which contribute to the emergence of antibiotic-resistant pathogens (Mancuso et al. 2021). This poses challenges in treating sepsis and other infections, leading to higher mortality rates and healthcare costs. Therefore, boosting host immunity could improve the resolution of infection more rapidly and prevent death.

Macrophages are pivotal components of the innate immune system and are responsible for recognizing and clearing invading pathogens (i.e., bacteria) through phagocytosis (Franken et al. 2016). This process is initiated when macrophages recognize and bind to bacteria *via* pathogen recognition receptors (PRRs), such as Toll-like receptors (TLRs) and C-type lectin receptors (CLRs), which detect pathogen-associated molecular patterns (PAMPs) on the surfaces of bacteria (Xiang and Fan 2010; Li et al. 2021). Additionally, bacteria can be opsonized by antibodies or complement proteins, enhancing their recognition by macrophage receptors such as Fc receptors and complement receptors (Bournazos et al. 2015). Upon receptor binding, actin polymerization is triggered, leading to the extension of pseudopodia that engulf bacteria into a membrane-bound vesicle known as a phagosome (Eisentraut et al. 2023). The phagosome then fuses with lysosomes to form a phagolysosome, where bacterial degradation occurs through the action of hydrolytic enzymes and reactive oxygen species (ROS) (Valenta et al. 2020). This phagocytosis procedure highlights the critical role of macrophages in host defense against bacterial infections.

Recently, bacterial extracellular vesicles (BEVs) have emerged as important mediators of communication between bacteria and host cells and are becoming a significant focus of research (Liu et al. 2024). BEVs are nano-sized membrane vesicles released by bacteria into the extracellular environment. These tiny lipid bilayer vesicles contain a diverse array of bioactive components, including proteins, nucleic acids, and lipids, and deliver them to host cells, thereby modulating host immune responses and inflammation processes (Yu et al. 2018; Liu et al. 2024). Notably, pathogenic bacteria such as *Helicobacter pylori* and *Pseudomonas aeruginosa* utilize BEVs to deliver virulence factors and disrupt host immune functions, exacerbating conditions such as gastritis and systemic inflammation (Ansari et al. 2019; Koeppen et al. 2021). In contrast, probiotics such as *Lactobacillus kefirgranum*, *Lactobacillus plantarum Q7*, and *Lactobacillus reuteri* produce BEVs that enhance intestinal barrier function and exhibit anti-inflammatory properties, making them promising candidates for therapeutic applications (Kang et al. 2020; Hao et al. 2021; Hu et al. 2021). Furthermore, probiotic extracellular vesicles (EVs) can influence the composition and activity of the gut microbiota, promoting the growth of beneficial bacteria while inhibiting the proliferation of harmful pathogens (Liang et al. 2022). This modulation helps to maintain a balanced microbial ecosystem, which is essential for immune function and overall health. In addition, probiotic EVs can stimulate the host immune system, enhancing immune cell activity and promoting the production of antimicrobial molecules such as cytokines and chemokines (Molina-Tijeras et al. 2019). They can influence the behavior of immune cells such as macrophages, dendritic cells, and T cells, to help maintain immune homeostasis (Morishita et al. 2022; Mandelbaum et al. 2023). By reducing inflammation, probiotic EVs may protect tissues from damage caused by excessive immune activation (Kim et al. 2018; Hao et al. 2021). Given their ability to modulate immune responses and maintain gut health, probiotic EVs offer a safer and potentially effective approach for preventing and treating inflammatory diseases and infections. However, knowledge about BEVs is still limited, and whether BEVs play a role in controlling infection during sepsis and the underlying molecular mechanism remain obscure.

Several recent studies have suggested that probiotic EVs can interact with macrophages and influence their functions. For example, probiotic EVs have been shown to modulate macrophage polarization, shifting macrophages toward the anti-inflammatory M2 phenotype, which is associated with tissue repair and resolution of inflammation (Kim et al. 2020). Additionally, probiotic EVs may stimulate the production of anti-inflammatory cytokines by macrophages, contributing to immune regulation and homeostasis (Hu et al. 2020). However, whether probiotic EVs affect macrophage phagocytosis to clear bacteria during sepsis has been less investigated. Herein, we performed an explorative study to determine the impact of probiotic bacteria-released EVs on macrophage phagocytosis and intracellular bactericidal ability in vitro and in vivo. Moreover, we examined the potential therapeutic effects of probiotic *Lactobacillus rhamnosus* Gorbach-Goldin (LGG)-derived EVs in sepsis using a feces-injection-in-peritoneum (FIP) model of polymicrobial sepsis. Our results implicate LGG-EVs as novel immunotherapeutic agents for the treatment of sepsis by promoting macrophage phagocytosis to eradicate bacteria.

## Materials and methods

### Bacterial strains and growth conditions

Three well-known probiotic strains *Lactobacillus rhamnosus* Gorbach-Goldin (LGG) and *Lactiplantibacillus plantarum* WCFS1 (LP WCFS1), were purchased from the American Type Culture Collection (ATCC, USA), and *Escherichia coli* Nissle 1917 (EcN) was kindly provided as a gift from Alison Weiss’s lab (University of Cincinnati). The LGG strain and LP WCFS1 strain were cultured in a screwed bottle containing MRS (De Man, Rogosa and Sharpe) broth supplemented with L-cysteine HCL (0.05%) and glycine (1%). The EcN strain was grown in a screwed bottle containing Luria-Bertani (LB) broth supplemented with L-cysteine HCL (0.05%) and glycine (1%). These bottles of bacteria were cultured at 37 °C under microaerobic conditions (sealed in a gas-generating pouch system plastic bag; Fisher Scientific, Inc. #B260685) with constant shaking at 100 rpm. The three different pH conditions (pH5, pH6.5, and pH8) for culturing LGG were adjusted by adding either hydrochloric acid or sodium hydroxide to the MRS broth. Bacterial growth curves were generated by sequential measurement of the OD at 660 nm using a spectrophotometer (Spectronic 20 Genesys, Thermo Fisher Scientific, MA, USA).

### Isolation and purification of BEVs

BEVs were isolated from the bacterial culture supernatant as described previously (Willms et al. 2016). In brief, bacteria were inoculated in complete MRS or LB medium at a ratio of 1:100 and cultured for 14–16 h until they reached the late log phase (OD660 of 0.8–1.0). Then, the bacteria were spun down at 8000×g for 10 min, and the supernatant was transferred to a fresh centrifuge-tube and spun again at 10,000×g for 20 min at 4 °C to completely remove any cellular debris. Next, the culture supernatant was passed through a 0.45-µm membrane (Corning, NY, USA) using a vacuum filtration device (Corning, NY, USA) to further remove large particles such as residual bacteria and cellular debris. After an additional filtration with a 0.22-µm membrane (Corning, NY, USA), the concentrate was then ultracentrifuged at 40,000×g for 3 h at 4 °C. The pellet was washed and resuspended in sterile phosphate-buffered saline (PBS; pH 7.4) and then further purified by ultracentrifugation at 40,000×g for 3 h at 4 °C again. The supernatant was carefully aspirated, and the precipitate (BEVs) was resuspended in PBS. The BEVs solution was used immediately or stored in aliquots at −80 °C.

### Characterization of BEVs

The size distribution of BEVs were determined using Nanoparticle Tracking Analysis (NTA) by a Zetasizer Nano ZS (Malvern Panalytical, UK). To maintain equal conditions, the BEVs’ fractions were diluted up to 1000-fold with sterile PBS in a way that 20–120 particles per frame were measured. The size distribution of BEVs was measured by Dynamic Light Scattering (DLS), and all the samples were evaluated in three replicates. The data were analyzed by Zetasizer software version 7.11 (Malvern Panalytical, UK).

### Bradford protein assay and lipoteichoic acid (LTA) assay

To verify the efficiency of BEVs’ purification and to quantify their protein content, Bradford protein assay was carried out using the protein assay dye reagent (Bio-Rad, USA). Add 10 µL of each standard protein solution or sample to separate wells of a microplate. Then, add 200 µL of the diluted Bradford reagent to each well, and mix gently. The plate was incubated at room temperature for 5–10 min to allow color development. The absorbance of each well was measured at 595 nm using a microplate reader (Tecan, Switzerland). A standard curve was plotted using the absorbance values of the standard protein solutions. The protein concentrations of the BEVs were calculated based on their absorbance values and the standard curve. The concentration of LTA in BEV^LGG^ was measured using an ELISA kit (MyBioSource, Cat. # MBS288308), following the manufacturer’s instructions. Optical density was measured at 450 nm, and LTA levels were determined from a standard curve.

### Cell culture

Raw264.7 macrophage cell line was purchased from ATCC. The cells were cultured in Dulbecco’s modified Eagle medium (DMEM, Cat. No.10-013-CV, NY, USA) supplemented with 10% fetal bovine serum (FBS) and 1% penicillin/streptomycin. The cells were cultured in an incubator at 37 °C with 5% CO_2_ and digested with 0.25% EDTA/trypsin every 2 or 3 days for cell passaging.

Bone marrow monocytes were isolated and cultured/differentiated into bone marrow-derived macrophages (BMDMs) as described previously (Wang et al. 2020). In brief, 6–8-week-old mice were terminally anesthetized, bone marrow from the tibias and femurs of the mice was flushed out using cold DMEM and filtered through 40 μm nylon cell strainer. Subsequently, these mononuclear cells were centrifuged at 1500×g for 5 min. The resulting cell pellet was resuspended in complete culture medium (DMEM supplemented with 15% L929 cell culture supernatant, 10% FBS, 1% penicillin/streptomycin solution), and cultured in a humidified atmosphere of 5% CO_2_ at 37 °C for 7 days, culture medium was changed on day 4 of culture.

### **Phagocytosis assay with *E. coli *BioParticles**

To assess the phagocytic capacity of Raw264.7 macrophages and BMDMs, the cells were seeded into 96-well plates (4 × 10^4^ cells per well or 5 × 10^4^ cells per well) in DMEM and allowed to adhere for 12 h. The cells were then treated with control (PBS with the same amount of LTA included in the BEVs, 3pg/mL), BEVs (10 µg/mL), N-tert-butyloxycarbonyl-Phe-Leu-Phe-Leu-Phe (Boc2, 10 µM) or its control DMSO (0.05%) for 12 h. After treatment, pHrodo red *E. coli* BioParticles (Invitrogen, Cat. # P35361) diluted in medium were added to each well according to the manuals and incubated for 1.5 h at 37˚C. The fluorescence intensity was measured using a Biotek Synergy H1 Hybrid Multi-Mode microplate reader (Bio Tek, USA). For the fluorescence microscopy assay, cells were seeded in dishes and allowed to adhere overnight. The cells were then fixed with 4% paraformaldehyde in PBS for 15 min at room temperature. Following fixation, the cells were permeabilized with 0.1% Triton X-100 in PBS for 10 min. To block non-specific binding, the cells were incubated with 5% bovine serum albumin (BSA) in PBS for 60 min. Subsequently, the cells were stained with CellMask Green Plasma Membrane Stain reagents (Invitrogen, Cat. # C37608) to label the cell membrane. After washing three times with PBS, the cell nuclei were stained with DAPI (Thermo Scientific, Cat. # 62248) at room temperature. Finally, a Leica stellaris 8 confocal microscopy (Leica Microsystems, Wetzlar, Germany) was used to capture images of the stained cells. Images were recorded with ZEN Black and analyzed with ImageJ software (Wayne Rasband, National Institutes of Health, MD, USA).

### Bacterial phagocytosis and killing assay

To determine bactericidal activity, a classical colony-forming unit (CFU) assay was conducted with minor modifications, as described previously (Mu et al. 2020). Briefly, *E. coli* DH5 alpha (New England Biolabs, Cat. # C29921) was grown overnight in LB at 37˚C. After that, the bacteria were quantified, pelleted, and washed with PBS. Raw264.7 macrophages or BMDMs were placed in 12-well culture plates (1 × 10^5^ cells per well or 2 × 10^5^ cells per well) and pre-incubated for 12 h. Subsequently, the cells were treated with control or BEV^LGG^ (10 µg/mL) in fresh medium for 12 h. Then, the medium was changed to antibiotic-free fresh medium, and after 0.5 h, the cells were infected with live *E. coli* at a multiplicity of infection (MOI) of 20 bacteria/cell ratio. After a 1-h incubation at 37˚C with 5% CO_2_, the infected macrophages were washed two times with cold PBS, and fresh DMEM containing 100 µg/mL gentamicin was added to incubate for 30 min to kill extracellular bacteria. To determine internal bacteria at this time, the cells were washed twice with PBS and lysed. The cell lysate was diluted serially and plated on LB plates to determine the CFU count. To assess the bactericidal activity, the cells were incubated for another 4 h. The number of internalized bacteria was determined by lysing the cells and plating the cell lysate, as described above. The killing percentage was calculated by using the following formula: [(CFU count at 30 min) -(CFU count at 4 h)/ (CFU count at 30 min)] ×100.

### Flow cytometry

Raw264.7 macrophages and BMDMs were seeded into 6-well plates (8 × 10^5^ cells/well or 1 × 10^6^ cells/well) in DMEM and allowed to adhere overnight. The cells were treated with control or BEV^LGG^ (10 µg/mL) for 12 h, followed by incubation with pHrodo red *E. coli* BioParticles for 1.5 h at 37 ^◦^C. Then, the cells were collected and fixed with 2% paraformaldehyde (15 min on ice). After the cells were washed with FACS buffer, flow cytometry analysis was performed. Analysis of 10,000 events gated on viable cells was performed on a BD LSRFortessa Cell Analyzer (BD, NJ, USA). The results were analyzed with FlowJo Software V10.10 (BD, NJ, USA).

### Mice

C57BL/6J mice were purchased from Jackson Laboratories (Bar Harbor, ME, USA). All mice were maintained and bred under specific pathogen-free conditions in the Division of Laboratory Animal Resources at the University of Cincinnati Medical Center. Male and female mice between 6 and 10 weeks of age were used for experiments in a gender-matched manner. All animal experiments conformed to the *Guide for the Care and Use of Laboratory Animals* prepared by the National Academy of Sciences, 2011, and published by the National Institutes of Health (NIH) and were approved by the University of Cincinnati Animal Care and Use Committee (#21-08-06-01).

### Mouse sepsis model induced by feces-injection-in-peritoneum (FIP), survival rate and murine sepsis score (MSS)

To simulate the clinical situation of polymicrobial sepsis more accurately, we used the FIP model to induce sepsis, as described previously (Cao et al. 2023). In brief, mice were euthanized, and the cecum containing the freshest feces was cut off with surgical scissors. The fresh feces were squeezed out from the cecum and transferred to a 50-mL tube and weighed on a scale. Then, the feces were mixed with sterile saline (0.9% NaCl) to a concentration of 40 mg/mL and stored at 4 °C for 24 h. To determine whether BEV^LGG^ has any therapeutic effects on polymicrobial sepsis in vivo, wild-type (WT) mice were anesthetized via intraperitoneal injection of a mixture of ketamine (100 mg/kg) and xylazine (5 mg/kg). The fecal mixture was subsequently injected intraperitoneally into the mice. Please note that, in our initial study, when the fecal supernatant was administered at 2 mg/g body weight to induce FIP model, we observed 100% mortality in the control group at 48 h. To reduce FIP-induced mortality in the vehicle group, we therefore decreased the fecal content-to-body weight ratio to 1.5 mg/g for this study. Mice were given sterile saline (1 mL) containing buprenorphine (4 µg/mL) subcutaneously immediately after fecal injection and then at 6-h intervals. BEV^LGG^ (2 µg/g body weight) or control (PBS with the same amount of LTA included in the BEVs, 0.6 pg/g body weight) was injected through the retro-orbital (R.O.) into the FIP-group 1 h after FIP. The sham-group received the same volume of sterile saline. To assess the actual effect of BEV^LGG^ on the survival of septic mice, antibiotics were not used in the FIP model. The animal survival rate was monitored every 6 h for 3 days after FIP. The MSS is a quantitative metric in which observations such as behavior, appearance, activity level, respiratory rate, and other physiological indicators are used to assess the severity of sepsis in mice and their response to septic insult (Mai et al. 2018). We monitored MSS every 2 h for 12 h after FIP. Whole blood and peritoneal lavage fluid (PLF) were harvested from these mice to measure bacterial burden, cytokine concentration, and biomarker levels of organ injury at 6 and 12 h post-FIP.

### Assessment of lung, liver, kidney injury and serum lactate dehydrogenase (LDH) levels

Lung tissues were collected from mice at 12 h post-FIP. All lungs were perfused *via* the heart, inflated, fixed with 10% buffered formalin, and then embedded in paraffin and cut into 5-µm sections. Tissue sections were stained with hematoxylin and eosin (H&E). To evaluate lung injury, 6–8 independent random fields per slide were evaluated for neutrophils in alveolar spaces, neutrophils in interstitial spaces, hyaline membranes, proteinaceous debris filling the airspaces, and alveolar septal thickening according to the relevance ascribed by the official American Thoracic Society workshop report on features and measurements of experimental acute lung injury in animals (Kulkarni et al. 2022).

Liver injury was determined by measuring the serum alanine aminotransferase (ALT) and aspartate aminotransferase (AST) concentration using an ALT assay kit (MyBioSource, Cat. # MBS264717) and an AST assay kit (MyBioSource, Cat. # MBS450720). Kidney damage was assessed by measuring the serum level of creatinine (Cr) using a Cr assay kit (MyBioSource, Cat. # MBS2504918), according to the manufacturer’s protocols. LDH levels in mouse serum were determined using a LDH ELISA kit (MyBioSource, Cat. # MBS720560) following the manufacturer’s instructions.

### Measurement of cytokines/chemokines and bacterial burden

Blood and PLF were collected at 6 h and 12 h after FIP. The levels of interleukin-6 (IL-6), tumor necrosis factor-α (TNF-α), interleukin-1β (IL-1β), and monocyte chemoattractant protein-1 (MCP-1) were measured in sera and PLF using commercially available ELISA kits [BioLegend, Cat. # 431301 (IL-6), Cat. # 430901 (TNF-α), Cat. # 432601 (IL-1β), Cat. # 432701 (MCP-1)], according to the manufacturer’s protocols.

The bacterial burden in the blood and PLF was measured as described previously (Wang et al. 2021). In brief, serial dilutions of peripheral blood or PLF from mice were cultured overnight on Casein Soybean Digest (CASO) agar plates (Cat #146004; Millipore-Sigma, USA) at 37 °C for 24 h, after which the bacterial CFUs were counted. In addition, we employed RT-qPCR to measure mRNA levels of cytokines/chemokines in lung tissue and bacterial 16 S rRNA levels in multiple tissue/organs collected from BEV-treated FIP mice and control mice.

### Isolation of RNA, RT-qPCR, RNA-seq, bioinformatic analysis, and gene set enrichment analysis (GSEA)

Total RNA was extracted from tissues and cells using the miRNeasy Mini Kit (QIAGEN, Germany) according to the manufacturer’s manual. cDNA was synthesized from 0.5 to 1.0 µg of RNA using SuperScript II Reverse Transcriptase (Invitrogen, USA). Then, quantitative real-time PCR was performed in triplicate with an Azure Cielo Real-Time PCR System (Azure Biosystems, Dublin, CA, USA) using SYBR Green mix (Radiant, USA). All primer sets used are listed in Supplementary Table S1. Gene expression was normalized to that of the reference gene glyceraldehyde-3-phosphate dehydrogenase (GAPDH), and the relative fold change in expression of the target genes was calculated by the 2^−ΔΔCt^ method. RNA-sequencing (RNA-seq) analysis was performed by Genomics, Epigenomics, and Sequencing Core at the University of Cincinnati using total RNA extracted from BMDMs after treatment with BEV^LGG^ and control for 12 h. The library was aligned and quantified using STAR V.2.6.1. FASTQ files were generated using bcl2fastq (Illumina). The reads were aligned to the mm10 genome assembly using the R subread (V.2.8.2). Counts were then summarized at the gene level using feature counts and normalized using Deseq2 (V.1.34.0). Differentially expressed genes (those with an absolute log2-fold change greater than 0 and an FDR-adjusted p value less than 0.1) were identified with DESeq2. GSEA was performed using GSEA software (v. 4.2.3) to compare gene expression signatures between BEV^LGG^-BMDMs and control-BMDMs. GSEA scores were calculated for sets with a minimum of ten detected genes, and all other parameters were set to their default.

### Reactive oxygen species (ROS) measurements

ROS levels were measured using a Cellular ROS assay kit (Abcam, Cat. # ab186027), according to the manufacturer’s instructions. Briefly, Raw264.7 macrophages and BMDMs (2 × 10^4^ cells per well or 4 × 10^4^ cells per well) were seeded into a 96-well plate at 70–80% confluency and incubated with the ROS Red Stain working solution provided in the kit for 1 h prior to treatment. Heat-inactivated *E. coli* solution (MOI = 20) was subsequently added to the wells. After a 30 min incubation, fluorescence was measured at 520 nm/605 nm using a Biotek Synergy H1 Hybrid Multi-Mode microplate reader (Bio Tek, USA). ROS levels were normalized to those of the control group.

### Western blot analysis

Western blot analysis was performed as described previously (Zhao et al. 2023). Total protein was extracted from cells using RIPA buffer supplemented with protein inhibitors (Thermo Scientific, Cat. # A32961), and the protein concentration was determined using Bradford protein assay. The protein samples were separated via 12% SDS-PAGE and subsequently transferred to PVDF membranes. 5% milk was used to block the membrane for 1 h, after which the membrane was washed thrice in Tris-buffered saline with tween 20 (TBST) (Fisher Scientific, Cat. # BP337-500). The membrane was incubated with primary antibodies at 4 °C overnight. After the membrane was washed three times with TBST, they were incubated with the secondary antibody for 1 h at room temperature. The bands were detected using WesternBright ECL (Advansta, Cat. # K-12049-D50) according to the manufacturer’s instructions. The protein gray values were calculated with Image J software (Wayne Rasband, National Institutes of Health, MD, USA). Primary antibodies, including Phospho-AKT1/AKT2/AKT3 polyclonal antibody (1:1000, Cat. # PA5-104445), AKT Pan polyclonal antibody (1:1000, Cat. # 44-609G), and GAPDH antibody (Cat. # MA5-15738), were purchased from Invitrogen (Waltham, MA, USA). HRP-Goat Anti-Rabbit IgG (H + L) (Invitrogen, Cat. # 656120) was used as a secondary antibody.

### Statistical analysis

All the statistical analyses were performed using GraphPad Prism version 10.0 (GraphPad Software, San Diego, CA). The data are presented as the mean ± standard error of the mean (SEM) of at least three independent experiments. A normal distribution was assessed with Kolmogorov-Smirnov test. To determine the statistical significance of differences among groups, one-way analysis of variance (ANOVA) followed by Tukey’s post hoc test was performed. A p-value of < 0.05 was considered indicate statistical significance. For the evaluation of the interaction between two independent variables, two-way ANOVA was conducted, followed by Bonferroni post hoc correction to identify specific group differences.

## Results

### Physical characterization of BEVs released by probiotic bacteria and their effects on macrophage phagocytosis

**Fig. 1:**
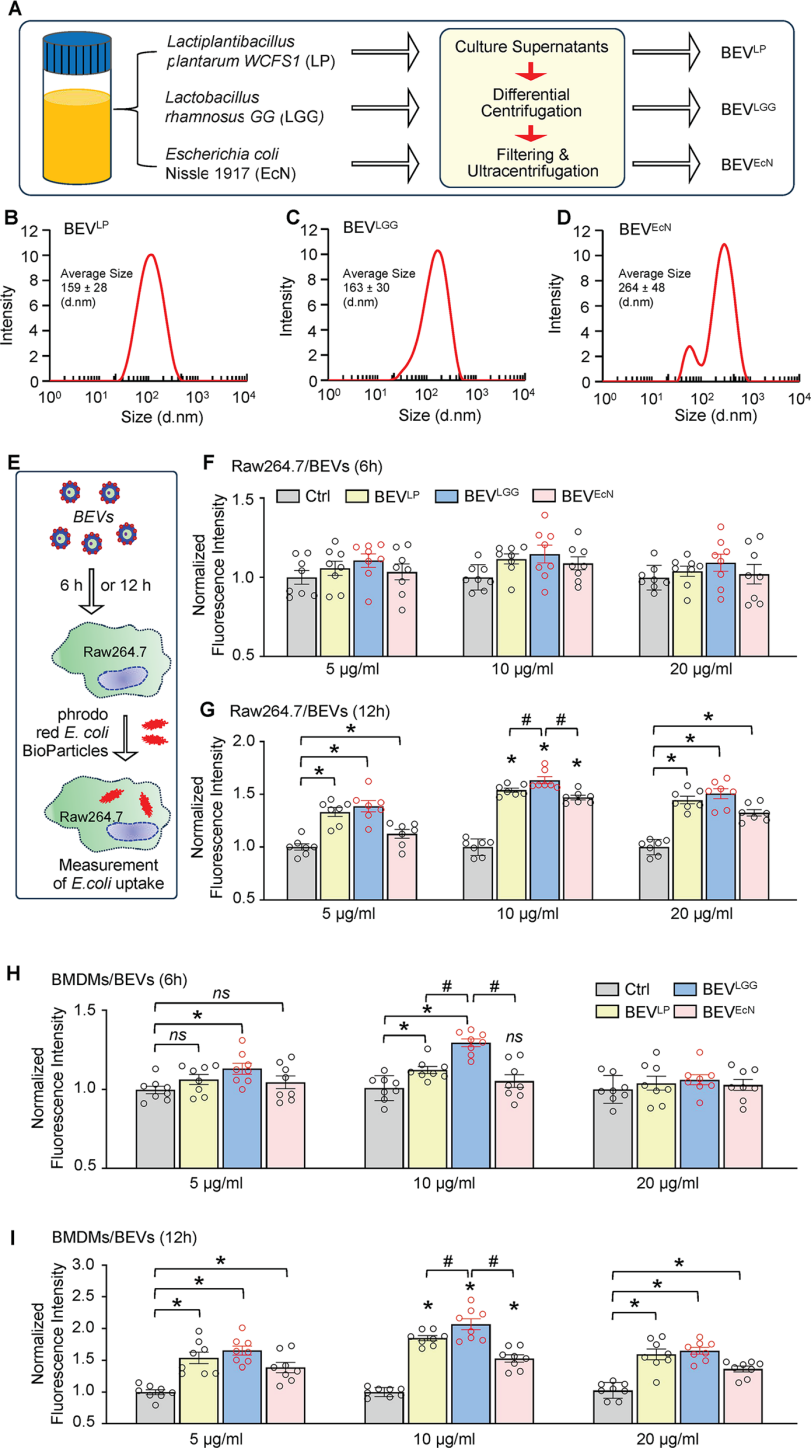
Physical characterization of BEVs released from probiotic bacteria and their effects on macrophage phagocytosis. **A** Flow chart for the collection and purification of BEVs from the cultivated probiotic bacteria. **B-D** Size distribution [**B**: LP WCFS1, **C**: LGG, **D**: EcN] of these BEVs determined by nanoparticle tracking analysis. **E** A cartoon scheme describing the procedure to measure phagocytic capacity in Raw264.7 macrophages after treatment with BEVs. **F-I** After incubation of Raw264.7 macrophages [**F**: 6 h, **G**: 12 h] and BMDMs [**H**: 6 h, **I**: 12 h] with BEVs derived from three probiotics at different doses for 6 h and 12 h, respectively, the phagocytic capacity was measured by determining the red fluorescence intensity with a plate reader. All results are presented as mean ± SEM and analyzed by 2-way ANOVA (*p < 0.05 vs. control group, ^#^*p* < 0.05 vs. BEV^LP^ and BEV^EcN^, ns: non-significance)

Given that LP WCFS1, LGG and EcN are three well-characterized probiotics, we cultured them under microaerobic conditions and collected the culture supernatants to isolate the bacteria-released extracellular vesicles (BEVs), as depicted in Fig. 1A. Using a protein concentration assay and NTA, we observed that the protein content of the BEV^LP^ preparations was approximately 0.91 µg/µl, and the average diameter of the BEV^LP^ was 159 ± 28 nm (Fig. 1B). Moreover, BEV^LGG^ contained 1.02 µg/µl protein with an average diameter of 163 ± 30 nm (Fig. 1C), and BEV^EcN^ contained approximately 1.17 µg/µl protein with an average diameter of 264 ± 48 nm (Fig. 1D).

Considering that bacteria can naturally stimulate macrophage phagocytosis, we next asked whether these bacteria-released EVs had a similar effect on macrophages. To this end, we initially performed bacterial phagocytosis assays using a cell line, Raw264.7 macrophages. As depicted in Fig. 1E, BEV^LP^, BEV^LGG^, or BEV^EcN^ were added to Raw264.7 cells at different doses (5, 10, and 20 µg/mL), and the cells were incubated for 6 h or 12 h. The phagocytic capacity was subsequently assessed by measuring the uptake of red fluorescent-conjugated pHrodo *E. coli* BioParticles. Interestingly, after incubating for 6 h, neither of these BEVs promoted the uptake of pHrodo *E. coli* BioParticles by Raw264.7 cells, as evidenced by the similar red fluorescence intensities among these BEV-treated cells and control cells (Fig. 1F). However, after the incubation time was extended to 12 h, all three BEV-treated Raw264.7 cells displayed a significantly higher intercellular red fluorescence intensity than did the control-treated cells (Fig. 1G). Further analysis of Raw264.7 cells revealed that incubating BEV^LGG^ at a dose of 10 µg/mL for 12 h resulted in greater phagocytic capacity than incubating either BEV^LGG^- or BEV^EcN^-treated cells under the same conditions (Fig. 1G). These results indicate that BEVs are capable of stimulating macrophage phagocytosis but have strain-, time-, and dose-dependent effects.

To further determine whether BEVs could play a similar role in ex vivo macrophages as in vitro cell line above, we isolated/cultured BMDMs and incubated them with these BEVs at the same doses and duration as those used for Raw264.7 cells. Unlike their effects on Raw264.7 cells, different bacterial strain-derived EVs have distinctive phagocytosis capacities on BMDMs. As shown in Fig. 1H, after incubation with 5 µg/mL BEVs for 6 h, only BEV^LGG^-treated BMDMs exhibited a significantly higher uptake of *E. coli* bioparticles, compared to control-treated cells. Importantly, BMDMs were treated with 10 µg/mL BEVs for 6 h, both BEV^LP^ and BEV^LGG^, but not BEV^EcN^, were able to augment macrophage phagocytosis compared to that in control-treated cells. (Fig. 1H). Notably, BEV^LGG^ could stimulate macrophage phagocytosis to a greater degree than BEV^LP^. Unexpectedly, when the BEV dose was increased to 20 µg/mL for 6 h, none of the three BEVs promoted macrophage phagocytosis (Fig. 1H). Consistently, when extended BEV-treatment time to 12 h, all BEVs could significantly enhance phagocytosis capacity in BMDMs, compared to control cells (Fig. 1I), similar to what was observed in the Raw264.7 cells (Fig. 1G). Notably, among the three bacterial strain-derived EVs, BEV^LGG^ had the greatest capacity to stimulate BMDMs phagocytosis at a dose of 10 µg/mL (Fig. 1I). Therefore, we selected probiotic LGG to generate BEVs and chose a dose of 10 µg/mL for the following in vitro studies.

### **Characterization of BEVs released from LGG cultured under acid**,** neutral**,** and alkaline conditions and their effects on macrophage phagocytosis**

**Fig. 2:**
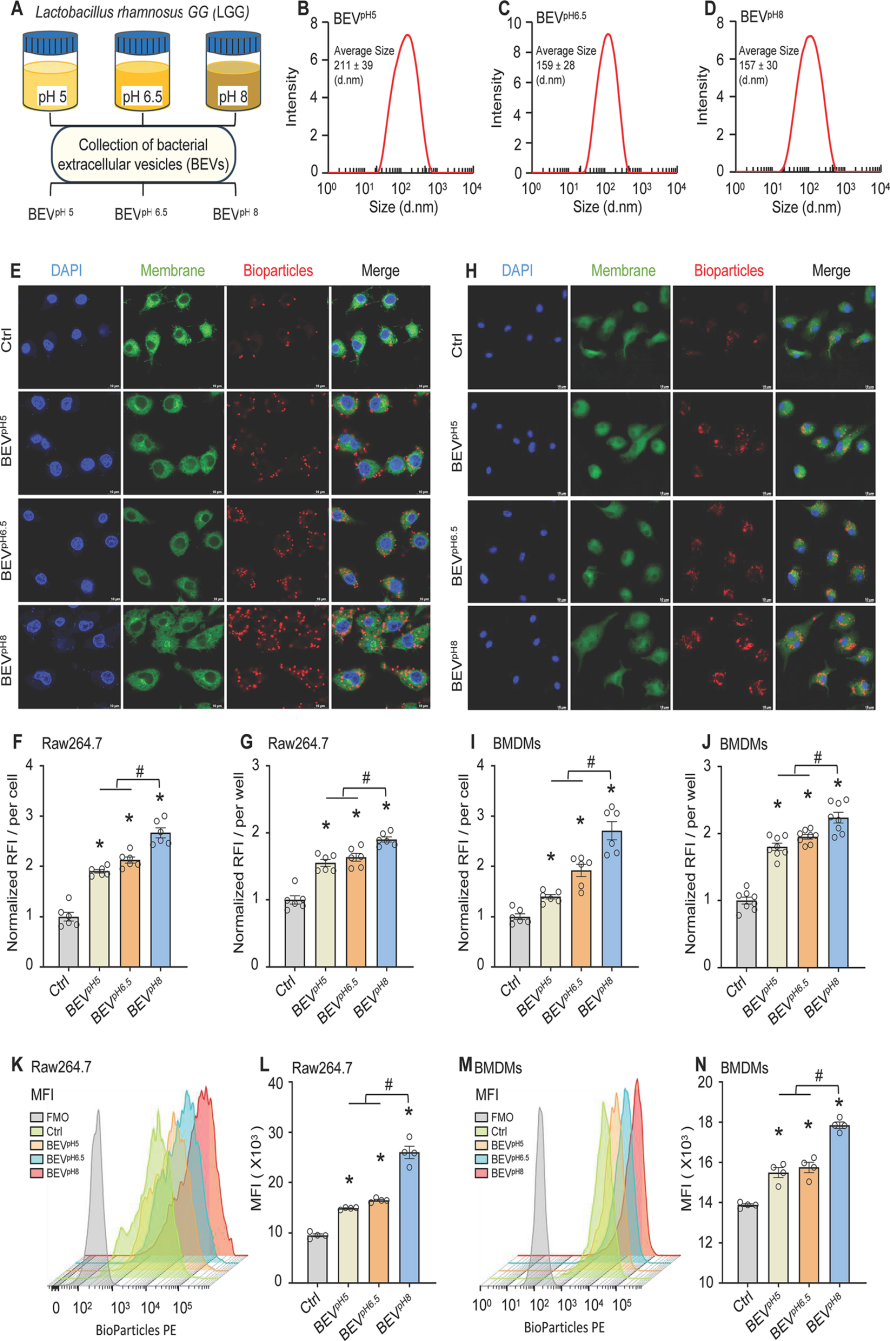
Physical characterization of BEVs released from LGG cultured under acidic, neutral, and alkaline conditions and their effects on macrophage phagocytosis. **A** Flow chart for the isolation of BEVs from LGG cultivated under three different pH conditions. **B-D** Size distribution [**B**: BEV^pH5^, **C**: BEV^pH6.5^, **D**: BEV^pH8^] of these BEVs determined by nanoparticle tracking analysis. **E-J** Representative confocal images of *E. coli* bioparticles engulfed by Raw264.7 macrophages **(E)** and BMDMs **(H)** after treatment with LGG-derived BEVs (10 µg/mL) (scale bar, 10 μm). The normalized mean fluorescence intensity in Raw264.7 macrophages **(F)** and BMDMs **(I)** was quantified. After incubation of Raw264.7 macrophages **(G)** and BMDMs **(J)** with BEVs derived from LGG cultured under three different pH conditions for 12 h, the phagocytic capacity was assessed by determining the red fluorescence intensity with a plate reader. **K-N** Representative flow cytometry histograms and median fluorescence intensity values showing phagocytosis of *E. coli* bioparticles by Raw264.7 macrophages **(K**,** L)** and BMDMs **(M**,** N)** treated with LGG-derived BEVs cultured under three different pH conditions. All results are presented as mean ± SEM and analyzed by one-way ANOVA (*p < 0.05 vs. control group, ^#^*p* < 0.05 vs. BEV^pH5^ and BEV^pH6.5^)

It has been reported that pH value, an important parameter of culture medium, directly affects bacterial growth, metabolism, and synthesis of bioactive substances (Wang et al. 2020; Zaghloul et al. 2023). Accordingly, the properties of BEVs collected from probiotics under different pH conditions may be distinctive. Along this line, we cultured LGG under acid (pH5), standard (pH6.5), and alkaline (pH8) conditions to generate BEV^pH5^, BEV^pH6.5^, and BEV^pH8^, as depicted in Fig. 2A. Firstly, we measured LGG growth rate and observed that three pH conditions did not significantly alter their growth curve (Supplementary Fig. S1A). Given that LTA is a major component of the cell wall of Gram-positive bacteria and plays a role in bacterial adherence, immune system activation, and inflammation, we then measured LTA levels in these BEVs. Unexpectedly, our results showed that the LTA contents in BEV^pH5^, BEV^pH6.5^, and BEV^pH8^ were very low and less than 0.45 pg/µg, and there were no statistical differences among the three groups of BEV^pH5^, BEV^pH6.5^, and BEV^pH8^ (Supplementary Fig. S1B). However, the average diameter size of BEV^pH5^ was significantly larger (211 ± 39 nm; Fig. 2B), compared to that of BEV^pH6.5^ (159 ± 28 nm; Fig. 2C) and BEV^pH8^ (157 ± 30 nm; Fig. 2D).

Next, we determined the effects of these BEVs on macrophage phagocytosis, using three different methods including: (1) confocal microscopy, (2) microplate reader, and (3) flow cytometry to measure fluorescence intensity of red pHrodo *E. coli* BioParticles taken up by macrophages. To exclude possible effects of LTA contained in the BEVs, we used PBS plus an equal amount of LTA that was included in the corresponding BEV^LGG^ as a control. As shown in Fig. 2E/F, treatment of Raw264.7 macrophages with BEV^pH5^, BEV^pH6.5^, or BEV^pH8^ for 12 h significantly increased the uptake of red *E. coli* bioparticles, compared with control cells. Importantly, the highest amount of red bioparticles were observed in the BEV^pH8^-treated Raw264.7 cells, compared to both BEV^pH5^- and BEV^pH6.5^-treated groups as determined via confocal microscopy. Consistently, using microplate reader, we found that the intracellular red fluorescence intensity was significantly higher in all BEV-treated Raw264.7 macrophages than control-treated cells with the highest in BEV^pH8^-treated group (Fig. 2G). Similar results were also observed in BMDMs upon exposure to these BEVs for 12 h, followed by addition of red pHrodo *E. coli* BioParticles and subsequently analyzed by confocal microscopy (Fig. 2H/I) and microplate reader (Fig. 2J). Additionally, flow cytometry analysis revealed higher red fluorescence content in all BEV-treated Raw264.7 macrophages (Fig. 2K/L) and BMDMs (Fig. 2M/N), compared to the control-treated cells, while the red fluorescence intensity was strongest in BEV^pH8^-treated macrophages (Fig. 2K–N). Taken together, these data consistently demonstrate that BEVs released by LGG bacteria, regardless of culture pH conditions, can greatly promote macrophage phagocytosis. Despite that, BEVs from LGG cultured in pH8-MRS medium are the best at stimulating macrophage phagocytosis.

### LGG-released BEVs enhance phagocytic and bactericidal activities of macrophages

**Fig. 3:**
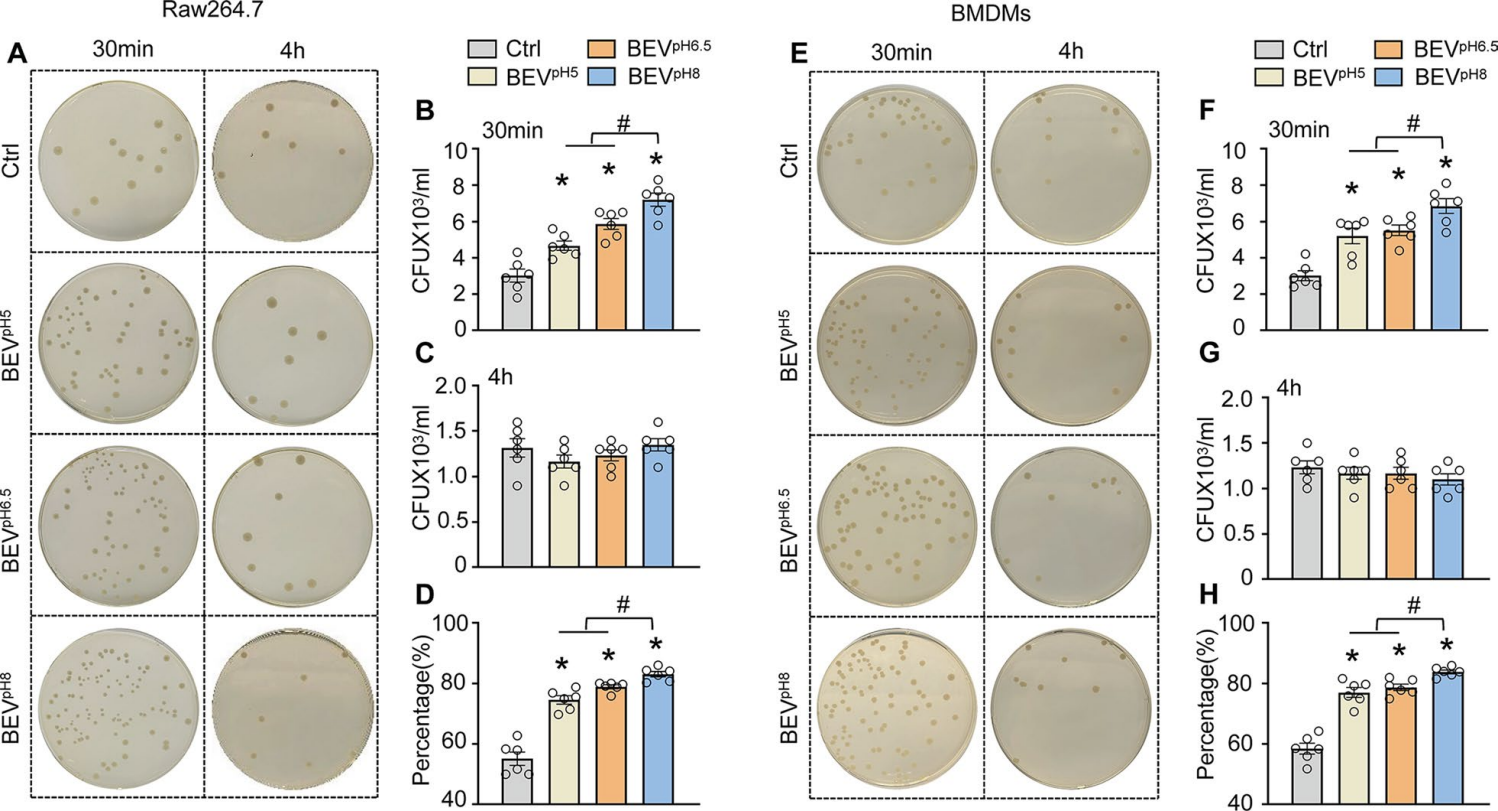
LGG-derived BEVs cultured under three different pH conditions can promote the phagocytic activity and intracellular bactericidal ability of macrophages toward live bacteria. Gentamicin protection assay was used to test phagocytic and bactericidal activities of Raw264.7 macrophages **(A)** and BMDMs **(E)** using live *E. coli*. Gentamicin (100 µg/mL) was added to the culture medium 1 h after infection with live *E. coli* (MOI = 20). After 30 min, the cell lysate was diluted using the serial dilution method and plated on LB agar plates. The CFUs were measured after 24 h as an indicator of phagocytosis capacity in Raw264.7 macrophages **(B)** and BMDMs **(F)**. In addition, CFUs in Raw264.7 macrophages **(C)** and BMDMs **(G)** lysates were determined 4 h after the addition of gentamicin to assess the number of bacteria remained within the macrophages. The killing percentages of Raw264.7 macrophages **(D)** and BMDMs **(H)** were calculated as described in the Materials & Methods section (*n* = 6). All results are presented as mean ± SEM and analyzed by one-way ANOVA (*p < 0.05 vs. control group, ^#^*p* < 0.05 vs. BEV^pH5^ and BEV^pH6.5^)

To further determine the effects of LGG-released BEVs on the control of bacterial dissemination, we next utilized live *E. coli* in place of fluorescence-conjugated bioparticles to infect BEV-treated macrophages, followed by bacterial uptake and intracellular killing assays. As shown in Fig. 3A/B, a greater number of bacteria was observed in all BEV-treated Raw264.7 cell lysates collected 30 min after bacterial infection, compared to the control group, while pre-treatment of Raw264.7 macrophages with BEV^pH8^ displayed the highest among three BEV-treated groups. These data further validate that LGG-BEVs can stimulate macrophages to take up bacteria with BEV^pH8^ to the greatest extent. At 4 h post-bacterial infection, we collected macrophage lysates and determined the number of bacterial retained in BEV-treated macrophages to calculate the percentage of bacteria killed according to the formula described in the Methods section. While there was no difference in the number of bacterial CFUs among the 4-h-cell lysates (Fig. 3A/C), the percentage of intracellular bacterial killing revealed that pre-treatment of Raw264.7 cells with BEV^pH8^ exhibited the highest bactericidal effects (83.1%), followed by pre-treatment with BEV^pH6.5^ (78.9%) and BEV^pH5^ (74.5%), all were better than control group (55.2%) (Fig. 3D). Similarly, BEV-treated BMDMs showed a significant increase in bacterial uptake and killing rate compared with control-BMDMs, meanwhile the most increase was observed in BEV^pH8^-treated BMDMs, compared to both BEV^pH5^- and BEV^pH6.5^-treated cells (Fig. 3E–H). Collectively, these data further indicate that probiotic LGG-released BEVs can remarkably enhance macrophage phagocytosis and bactericidal activity. Particularly, BEVs collected from pH8-condition LGG demonstrated the highest potency in promoting bacterial clearance in macrophages. Therefore, we selected BEV^pH8^ to test their therapeutic effects in septic mice (below).

### Administration of BEV^pH8^ attenuates polymicrobial sepsis-induced mortality and organ injury in mice

**Fig. 4:**
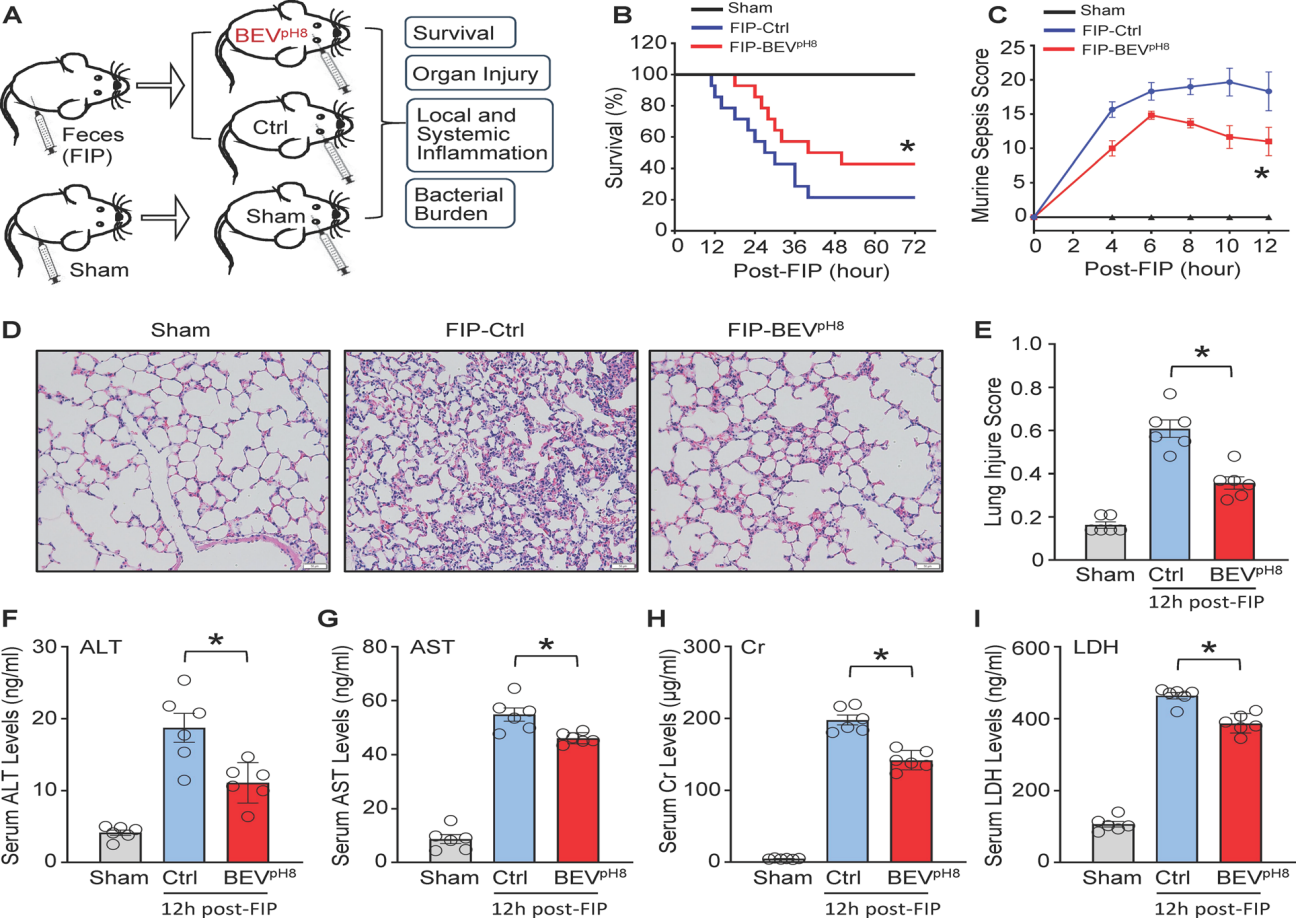
Administration of BEV^pH8^attenuates polymicrobial sepsis-induced mortality and organ injury in mice. **A** Graphic illustration of experimental design: Feces-injection-in-peritoneum (FIP)-induced sepsis mouse model, and administration of BEV^pH8^ (2 µg/g), or control through the retro-orbital (R.O.) injection at 1 h post-FIP. **B** Kaplan–Meier survival curves were generated to compare mortality between groups, and significance was determined by log-rank (Mantel-Cox) test (*p < 0.05). **C** The murine sepsis score (MSS) was assessed at various time points by observing and recording behavioral, visual, activity levels, respiratory rate, and other physiological parameters of the mice (*p < 0.05). **D** Representative images of lung sections stained with hematoxylin and eosin (H&E) from sham, control-, and BEV^pH8^-treated mice at 12 h post-FIP (scale bar, 50 μm). **E** The lung injury scores were assessed as described in the Materials & Methods section (*n* = 6). **F–I** Serum levels of ALT **(F)**, AST **(G)**, Cr **(H)**, and LDH **(I)** in each group were measured at 12 h post-FIP using ELISA kits (*n* = 6). All results are presented as mean ± SEM and analyzed by 2-way ANOVA (*p < 0.05 when compared with respective control group)

To investigate the in vivo therapeutic effects of BEV^pH8^, we employed a feces-injection-in-peritoneum (also named fecal-induced peritonitis, FIP) model of murine sepsis, which is closely mimicked the clinical conditions (Sharma et al. 2023) and more suitable for standardization than cecal ligation and puncture (CLP)-sepsis model (Fang et al. 2020). At 1-h post-FIP, BEV^pH8^ (2 µg/g body weight) or control (PBS plus the same amount of LTA included in the BEVs, 0.6 pg/g body weight) was administered *via* R.O. injection followed by monitoring for mortality over a 3-day period and other experimental analysis (Fig. 4A). We observed that BEV^pH8^-treated mice exhibited a significantly higher survival rate than control-treated mice did over a period of 3 days post-FIP (Fig. 4B). Additionally, we recorded the MSS, a quantitative metric in which behavior, appearance, activity level, respiratory rate, and other physiological indicators are scored. We found that sepsis scores at various time points were lower in the BEV^pH8^-treated group compared to the control group (Fig. 4C). This suggests that the control group exhibited more severe sepsis-related symptoms and organ dysfunction after FIP. Notably, there was a decreasing trend in the murine sepsis scores in the BEV^pH8^-group starting at 8 h post-FIP, indicating effective control of the bacterial infection. Furthermore, we observed that FIP-induced lung injury was significantly mitigated in BEV^pH8^-treated group, as evidenced by the greater decrease in neutrophil infiltration, formation of hyaline membranes, thickness of alveolar wall, and the alveolar collapse in lung tissues collected from BEV^pH8^-injected mice (injury score: 0.358 ± 0.064), compared to those from control-treated mice (injury score: 0.608 ± 0.091) (Fig. 4D/E). Next, we measured the serum levels of alanine aminotransferase (ALT, biomarker of liver injury), aspartate aminotransferase (AST, biomarker of liver and heart injury), creatinine (Cr, biomarker of kidney injury), and lactate dehydrogenase (LDH, biomarker of tissue damage) in BEV-treated mice at 12 h post-FIP, in comparison with control-treated septic mice. As shown in Fig. 4F–I, the serum concentrations of these biomarkers were significantly decreased in BEV^pH8^-treated mice, compared to control mice. Collectively, these data suggest that the administration of BEV^pH8^ could significantly rescue FIP-induced multi-organ injury in mice.

### Administration of BEV^pH8^ suppresses inflammatory response and reduces bacterial burden in FIP-septic mice

**Fig. 5:**
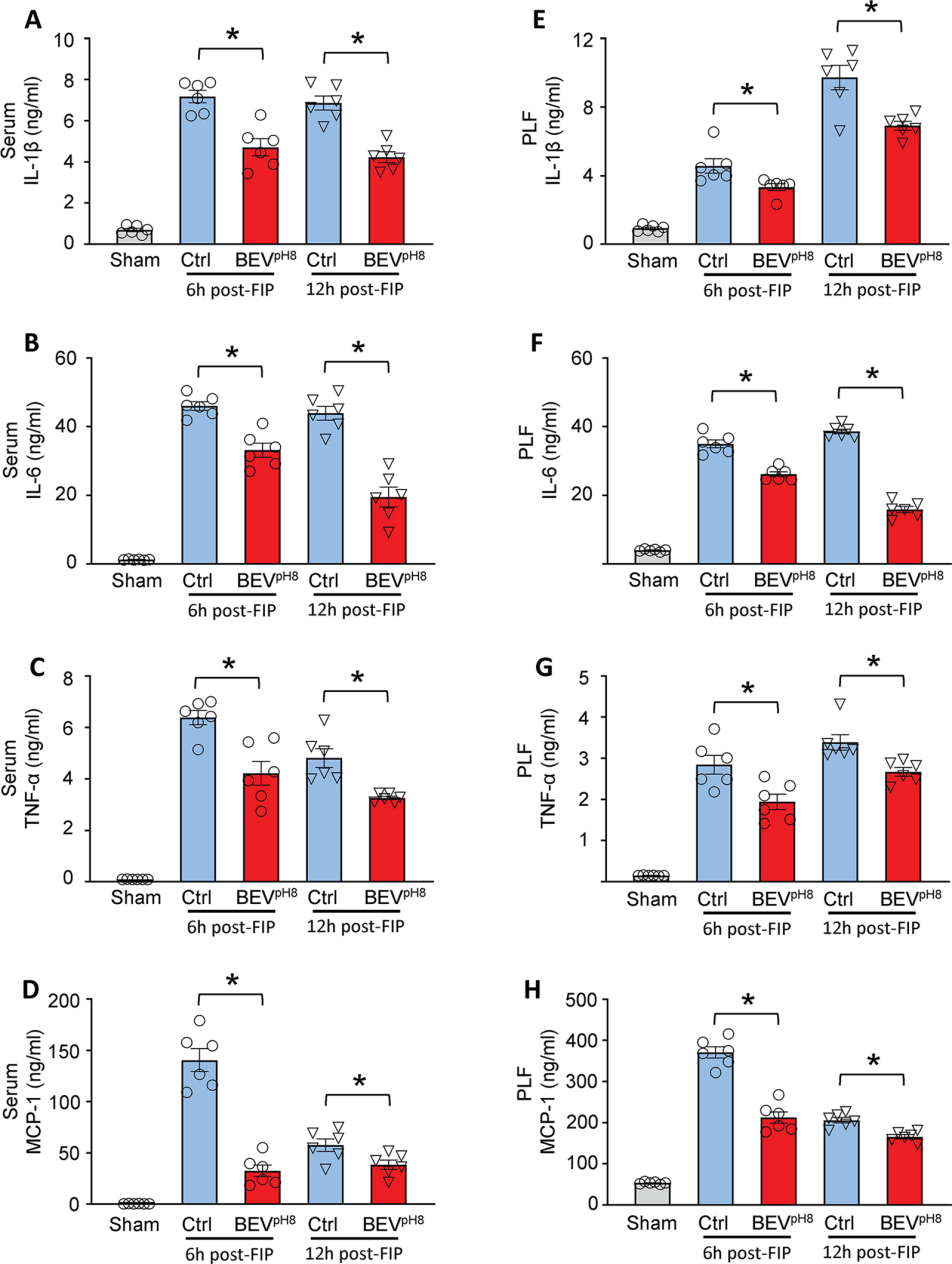
Administration of BEV^pH8^ mitigates both local and systemic inflammatory responses **A**,** E** IL-1β, **B**,** F** IL-6, **C**,** G** TNF-α, and **D**,** H** MCP-1 levels in the serum and PLF of Sham, control- and BEV^pH8^-treated mice at 6 h and 12 h post-FIP. All results are presented as mean ± SEM and were compared to respective control group at each time point by 2-way ANOVA (*n* = 6, *p < 0.05 when compared with respective control group)

Given that bacterial burden and inflammatory responses are important driving factors for the sepsis-triggered organ injury, we then collected peripheral blood and PLF from septic mice at 6 h and 12 h after FIP to determine the impact of BEV^pH8^ on the systemic and local inflammatory response and bacterial clearance. Using ELISA kits, we measured the concentrations of inflammatory cytokines and chemokines such as IL-1β, IL-6, TNF-α, and MCP-1, in the blood and PLF collected above. Our results show that the serum levels of IL-1β (Fig. 5A), IL-6 (Fig. 5B), TNF-α (Fig. 5C), and MCP-1 (Fig. 5D) were significantly lower in BEV^pH8^-treated mice than in control-treated mice at 6 h post-FIP and were more pronounced at 12 h post-FIP. Similarly, BEV^pH8^-mediated suppression of inflammatory response was also observed in the PLF (Fig. 5E–H). Additionally, we assessed mRNA levels of inflammatory cytokines and chemokines in lung tissues at 12 h post-FIP. Our results demonstrated that mRNA levels of IL-1β (Supplementary Fig. S2A), IL-6 (Supplementary Fig. S2B), TNF-α (Supplementary Fig. S2C), and MCP-1 (Supplementary Fig. S2D) in the lungs of BEV^pH8^-treated group were significantly lower, compared to the control group.

**Fig. 6:**
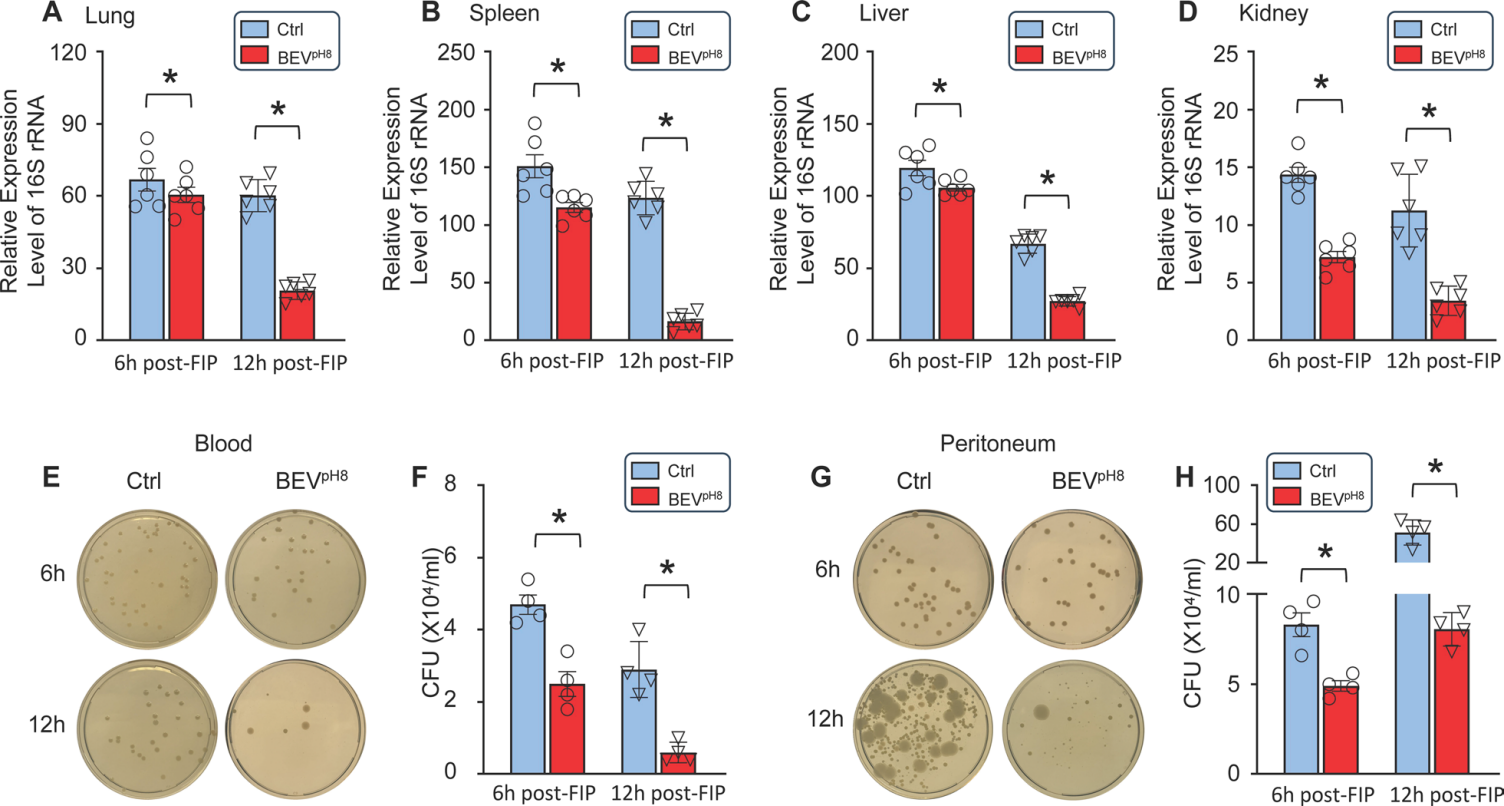
Administration of BEV^pH8^ decreases bacterial burden in multiple organs, blood, and PLF after FIP challenge. **A–D** Relative expression level of bacterial 16S rRNA in organs [**A**: Lung, **B**: Spleen, **C**: Liver, **D**: Kidney] of control- and BEV^pH8^-treated mice at 6 h and 12 h post-FIP by RT-qPCR (*n* = 6). **E–H** The bacterial burden in both blood **(E**,** F)** and PLF **(G**,** H)** were measured in control- and BEV^pH8^-treated mice at 6 h, and 12 h post-FIP. All results are presented as mean ± SEM and analyzed by 2-way ANOVA (*p < 0.05 when compared with respective control group)

Next, we evaluated bacterial clearance in various organs, including the lung, spleen, liver, kidney, and heart, by measuring the expression levels of 16S rRNA, a marker of bacterial presence. We detected that the levels of bacterial 16S rRNA were significantly decreased in the lungs (Fig. 6A), spleen (Fig. 6B), liver (Fig. 6C), and kidneys (Fig. 6D), collected from BEV^pH8^-treated mice, compared to those organs from control-treated mice at 6 h post-FIP, which were more dramatically reduced at 12 h post-FIP (Fig. 6A–D). However, there was much less bacterial 16S rRNA detected in the heart which showed no significant difference between two groups at either 6 h or12 h post-FIP (Supplementary Fig. S3A). Lastly, the bacterial load was examined in the blood and PLF of septic mice. We observed that bacterial burden was remarkably reduced in both blood (Fig. 6E, F) and PLF (Fig. 6G, H) collected from BEV^pH8^-treated mice in comparison with control-treated mice at 6 h and 12 h post-FIP. It is important to note here that the bacterial load was highly increased in the PLF compared to the blood, particularly at 12 h post-FIP. This phenomenon could be attributed to the feces-injection *i.p*. where bacteria continue to proliferate to peak levels in the peritoneal cavity at 6–12 h after FIP. Taken together, these data indicate that BEV^pH8^ has the strong potential to limit both systemic and local inflammatory responses as well as bacterial dissemination during sepsis.

### RNA sequencing analysis of gene expression profiles of BEV-treated BMDMs

**Fig. 7:**
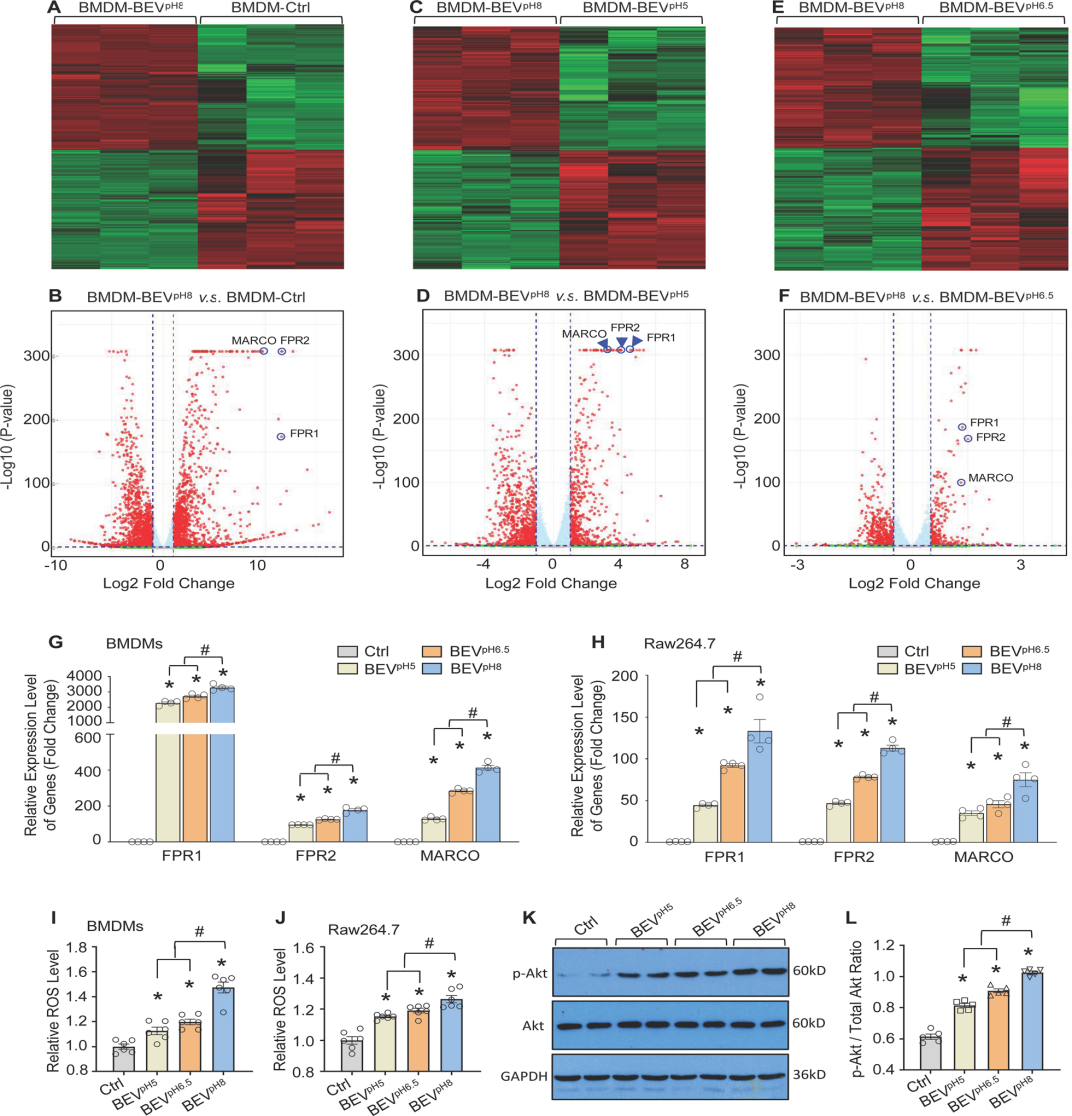
Gene expression profile in BMDMs treated with BEVs collected from the LGG cultures by high-throughput RNA sequencing. **A–F** Heatmap and volcano plot of the differentially expressed genes in BMDMs treated with control, BEV^pH5^, BEV^pH6.5^ or BEV^pH8^ [**A**,** B**: BEV^pH8 ^vs. control; **C**,** D**: BEV^pH8^ vs. BEV^pH5^; **E**,** F**: BEV^pH8^ vs. BEV^pH6.5^] (*n* = 3). The expression of FPR1, FPR2, and MARCO were among the most significantly altered genes in BEV-treated BMDMs, which were further validated in BMDMs **(G)** and Raw264.7 macrophages **(H)** by RT-qPCR (*n* = 6). **I**,** J** ROS levels were measured in BMDMs **(I)** and Raw264.7 macrophages **(J)** upon treatment with BEV^LGG^ for 12 h, followed by incubation with heat-killed *E. coli* for 30 min. **K**,** L** BMDMs were pretreated with control, BEV^pH5^, BEV^pH6.5^ or BEV^pH8^ (10 µg/mL) for 12 h. Then, cells were collected for Western blot analysis of total and phosphorylated Akt. Representative immunoblots **(K)** and their quantification analysis results **(L)** showing Akt activation. GAPDH was used as a loading control (*n* = 5). All results are presented as mean ± SEM and analyzed by one-way ANOVA (*p < 0.05 vs. controls, ^#^*p* < 0.05 vs. BEV^pH5^ and BEV^pH6.5^)

To elucidate the potential mechanisms by which LGG-derived BEVs enhance macrophage phagocytosis of bacteria, we treated BMDMs with BEV^pH5^, BEV^pH6.5^, BEV^pH8^, or control for 12 h, followed by isolating total RNAs for RNA-seq. Given that BEV^pH8^ has the strongest capacity in stimulating macrophage phagocytosis, then we plotted heatmaps and volcano plots for three comparisons as BEV^pH8 ^vs. Control (Fig. 7A/B), BEV^pH8 ^vs. BEV^pH5^ (Fig. 7C/D), and BEV^pH8 ^vs. BEV^pH6.5^ (Fig. 7E/F). Interestingly, among the top 30 differentially expressed genes, we were surprised to find that only three macrophage receptor genes including FPR1, FPR2, and MARCO were dramatically and commonly upregulated in BEV^pH8^-treated BMDMs, compared to control-, BEV^pH5^-, and BEV^pH6.5^-cells, respectively (Fig. 7A–F). FPR1 and FPR2 are formyl peptide receptors (FPRs) that are expressed at low levels on the surface of macrophages under basal conditions, while they can recognize bacterial peptides and initiate phagocytosis (He and Ye 2017; Liang et al. 2020). The macrophage receptor with collagenous structure (MARCO), a scavenger receptor, facilitates the recognition and uptake of microbial pathogens by macrophages (Zhou et al. 2024). Therefore, we selected these three receptor genes for validation via RT-qPCR. As shown in Fig. 7G, treatment of BMDMs with BEV^pH5^, BEV^pH6.5^, and BEV^pH8^ markedly upregulated the expression of FPR1, FPR2, and MARCO *by* > 2200-fold, > 96-fold, and > 128-fold, respectively, compared to control-treated control cells. Notably, BEV-mediated elevation of FPR1, FPR2, and MARCO expression was more pronounced in BEV^pH8^-BMDMs than in both BEV^pH5^- and BEV^pH6.5^-treated groups (Fig. 7G). Similar findings were also observed in Raw264.7 macrophages treated with BEV^pH5^, BEV^pH6.5^, and BEV^pH8^ (Fig. 7H).

Currently, it is well recognized that upregulation of FPR1/FPR2 can activate downstream signaling cascades, including: (1) Nicotinamide adenine dinucleotide phosphate (NADPH) oxidase, which induces ROS generation to facilitate bacterial killing inside macrophages (Xu et al. 2016); and (2) PI3K-Akt which can promote MARCO expression (Sun et al. 2023). Therefore, we next measured ROS levels in these BEV-treated macrophages upon stimulation with heat-killed *E. coli*. Our results showed that BEV^pH8^-treated BMDMs contained the highest levels of ROS, compared with other two BEV-treated groups, while all BEV-treated cells had higher levels of ROS than control-treated cells (Fig. 7I). Similar phenomena were also found in Raw264.7 macrophages treated with these LGG-derived BEVs (Fig. 7J). Regarding the PI3K-Akt pathway, we assessed Akt activity by measuring its phosphorylation in BEV-treated or control-treated BMDMs, using Western-blotting analysis. As shown in Fig. 7K/L, the phosphorylated Akt levels were significantly increased in all BEV-treated groups with the highest in BEV^pH8^-treated BMDMs, compared to control-treated BMDMs. Collectively, these data consistently indicate that BEVs released by probiotic LGG, especially cultured under pH8 condition can stimulate macrophages to strongly upregulate FPR1/2 expression, leading to increased ROS production and Akt activity as well as MARCO expression.

### Inhibition of FPR1/2 attenuates BEV^pH8^-mediated effects on macrophage phagocytosis

**Fig. 8:**
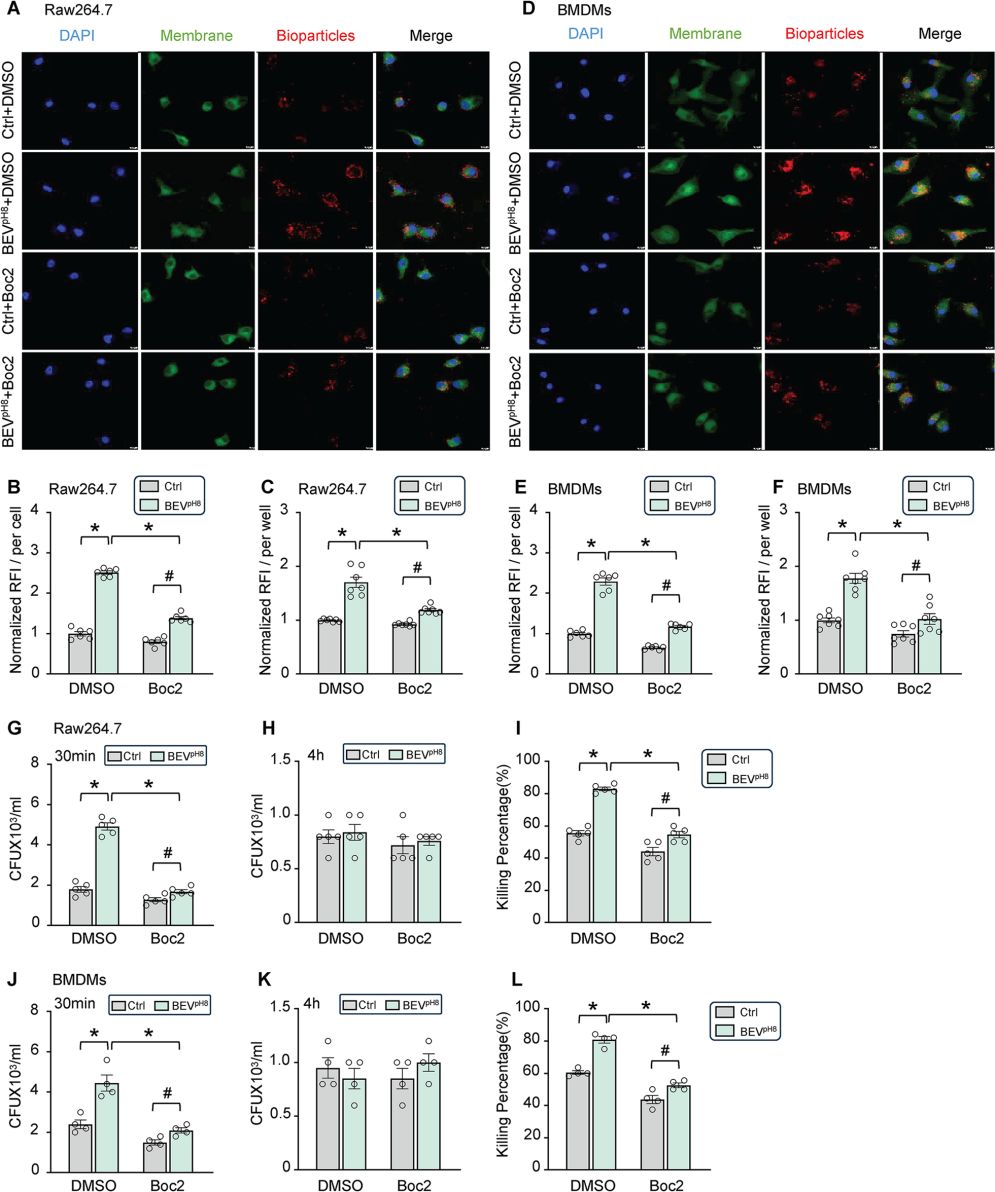
Inhibition of FPR1/2 abolishes BEV^pH8^-mediated effects on macrophage phagocytosis and bacterial killing activities. Raw264.7 macrophages (**A**-**C**) and BMDMs (**D**-**F**) were pre-treated with Boc2 (N-tert-butyloxycarbonyl-Phe-Leu-Phe-Leu-Phe), a specific antagonist of FPR1/FPR2 for 2 h, followed by incubation with BEV^pH8^ for 12 h. Then phagocytic capacity was assessed by the uptake of red *E. coli* bioparticles. Representative confocal images of phagocytosis of *E. coli* bioparticles by Raw264.7 macrophages **(A)** and BMDMs **(D)** (scale bar, 10 μm). The normalized mean fluorescence intensity in Raw264.7 macrophages **(B)** and BMDMs **(E)** was quantified. The phagocytic capacity of Raw264.7 macrophages **(C)** and BMDMs **(F)** was assessed by measuring the red fluorescence intensity using a plate reader. Gentamicin protection assay was used to detect the phagocytic ability and bactericidal activities in these Raw264.7 macrophages **(G-I)** and BMDMs **(J-L)** after treatment as described above. Gentamicin (100 µg/mL) was added to the culture medium 1 h after infection with live *E. coli* (MOI = 20). After 30 min, cell lysate was extracted with serial dilution, then plated on LB agar plates. The CFUs were measured as an indicator for phagocytosis capacity of Raw264.7 macrophages **(G)** and BMDMs **(J)**. CFUs were determined in lysates of Raw264.7 macrophages **(H)** and BMDMs **(K)** at 4 h after the addition of gentamicin to assess the number of bacteria remained within macrophages. The killing percentages of Raw264.7 macrophages **(I)** and BMDMs **(L)** were calculated as described in the Materials & Methods section (*n* = 6). All results are presented as mean ± SEM and analyzed by 2-way ANOVA (*p < 0.001, ^#^*p* < 0.05)

Lastly, we asked whether BEV^pH8^-mediated augmentation of macrophage phagocytosis is dependent on the upregulation of FPR1/2. To address this question, we pre-treated macrophages (Raw264.7 cells and BMDMs) with Boc2, a specific antagonist of FPR1/2, for 2 h, followed by incubation with BEV^pH8^ for 12 h and subsequently, macrophage phagocytosis analysis was performed using red pHrodo *E. coli* BioParticles. As shown in Fig. 8A, B, BEV^pH8^-treated Raw264.7 cells took up a remarkably higher amount of red *E. coli* particles than control-treated cells, which is consistent with the above data in Fig. 2E, F. However, BEV^pH8^-mediated dramatic increase of phagocytosis was greatly attenuated by pre-treatment of Raw264.7 cells with FPR1/2 inhibitor, Boc2; albeit it remained significantly higher than control cells (Fig. 8A, B). These results were further validated by measuring red fluorescence intensity using 96-well plates (Fig. 8C). Together, our data suggest that BEV^pH8^-mediated augmentation of phagocytosis is dependent on FPR1/2, at least in part. Similar outcomes were also shown in BMDMs upon pre-treated with Boc2, followed by addition of BEV^pH8^ for 12 h (Fig. 8D–F). Accordingly, further analysis using live bacterial infection revealed that the CFU counts from cell lysates of BEV^pH8^-treated Raw264.7 cells at 30-min post-infection were increased by 1.7-fold than controls in the absence of Boc2, but only increased by 0.3-fold than controls in the presence of Boc2 (Fig. 8G and Supplementary Fig. S4A). This suggest that BEV^pH8^-mediated uptake of bacteria is greatly blocked in macrophages when FPR1/2 are inhibited by Boc2. Although there were no difference in the CFU counts from all groups of cell lysates collected at 4-h post-infection (Fig. 8H), the percentage of intracellular bacterial killing was remarkably higher in BEV^pH8^-treated Raw264.7 cells, compared to control cells without pre-inhibition of FPR1/2 by Boc2 (Fig. 8I), but such BEV^pH8^-induced increase of bacterial killing rate was significantly diminished in Raw264.7 cells upon pre-treatment with Boc2 (Fig. 8I). In addition, similar inhibitory effects of Boc2 on BEV^pH8^-induced phagocytosis and bacterial clearance were also observed in BMDMs (Fig. 8J-L and Supplementary Fig. S4B). Collectively, our results demonstrate that inhibition of FPR1/2 could largely attenuate BEV^pH8^-mediated boost effects on macrophage phagocytosis and bactericidal activity.

## Discussion

**Fig. 9:**
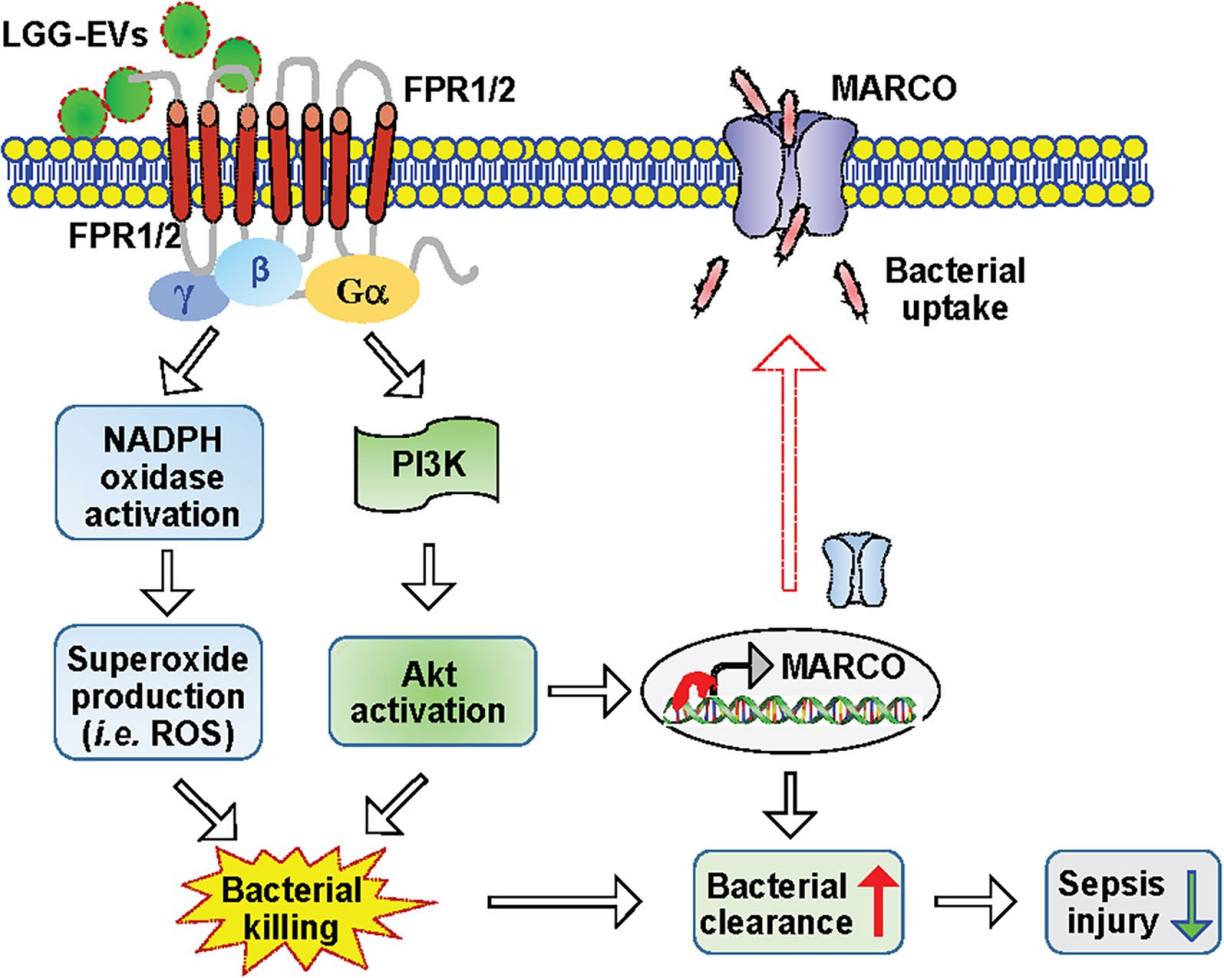
Scheme depicting that BEVs released by probiotic LGG could activate FPR1/2 and their downstream signaling pathways including NADPH-ROS and PI3K-Akt-MARCO, leading to increased uptake and clearance of bacteria and thereby reducing sepsis-induced organ injury and mortality

In the present study, we demonstrated that probiotic bacterial LGG-derived EVs exhibited the greatest effects among the three probiotic-derived BEVs on boosting macrophage phagocytosis and intracellular bactericidal activity, especially when BEVs were collected from LGG bacteria cultured in the pH8-alkaline medium. Therapeutically, in vivo administration of BEV^pH8^ to septic mice effectively reduces bacterial burden, attenuates systemic/local inflammatory responses, alleviates organ injury and eventually improves animal survival. Furthermore, RNA-seq and bioinformatic analysis revealed that LGG-BEVs could remarkably upregulate the expression of FPR1 and FPR2 in macrophages and thereby activate downstream signaling pathways (i.e., NADPH-ROS and PI3K-Akt-MARCO), leading to increased bacterial uptake and clearance (Fig. 9). Finally, we validated that BEV^pH8^-mediated increase in phagocytic and bactericidal activity could be partially antagonized by the FPR1/2 inhibitor, Boc2. Hence, our study clearly elucidates that probiotic BEVs could be a novel and effective therapeutic agent for the treatment of sepsis.

Currently, it has been recognized that, in sepsis patients, macrophages exhibit impaired phagocytosis, leading to ineffective eradication of invading bacteria (Yang et al. 2019). Consequently, the remaining bacteria can cause persistent local infection, which may further progress to systemic inflammation (Hortová-Kohoutková et al. 2020). As a matter of fact, approximately 80% of patients with sepsis still present with unresolved septic focus at the time of death, despite appropriate antibiotic therapy (Torgersen et al. 2009). Therefore, therapeutic strategies aimed at enhancing macrophage phagocytosis and bactericidal activity may have great potential for improving patient survival. While bacteria can serve as natural stimuli to boost macrophage activation and phagocytosis (Mu et al. 2021; Liu et al. 2024), the use of live non-pathogenic bacteria in therapeutic settings can pose risks. Over the past few decades, probiotic bacteria and their derived metabolites/supernatants have been well characterized to exert beneficial effects in various infection models (He et al. 2017; Khodaii et al. 2017). For example, He et al. (2017) reported that the culture supernatant from *L. rhamnosus* GG induces resistance to *E. coli* K1 infection by enhancing intestinal defense in neonatal rats. Similarly, Khodaii et al. (2017) showed that the supernatants of *Lactobacilli* and *Bifidobacteria* effectively protect enterocyte cell lines from invasion by enteroinvasive *E. coli*. Nevertheless, the underlying mechanisms remain unclear. Recently, several studies have demonstrated that probiotics can actively release EVs, which may be a critical contributor to these beneficial effects induced by probiotic supernatants. Interestingly, the present study further elucidated the novel role of probiotic EVs in boosting macrophage phagocytosis to efficiently clear bacteria in vitro and in vivo. Thus, probiotic EVs could be an excellent reagent in place of live bacteria to stimulate macrophage phagocytosis.

It is important to note here that different strains of bacteria can yield distinctive BEVs based on their physical, biochemical, and physiological properties (Liu et al. 2024). For example, BEVs released by the probiotic *Propionibacterium freudenreichii* strain exhibit anti-inflammatory activity in HT-29 human intestinal epithelial cells mostly *via* the EV surface protein [i.e., surface-layer protein B (SlpB)] (Rodovalho et al. 2020). However, BEVs derived from *Porphyromonas gingivalis*, *Treponema denticola*, and *Tannerella forsythia* could stimulate pro-inflammatory responses in macrophages/monocytes with the greatest effects being caused by *Tannerella forsythia-*BEVs and the least effects being caused by *Porphyromonas gingivalis-*BEVs (Cecil et al. 2017). In the present study, BEVs were collected from three well-known probiotic strains including two gram-positive strains (LGG and LP WCFS1) and one gram-negative strain, EcN 1917 and showed that the average diameter size of EcN-BEVs was significantly larger than those from bacterial strains of LGG and LP WCFS1. In addition, their effects on macrophage phagocytosis are also exhibited distinctive with the highest capacity by LGG-derived BEVs. Furthermore, several recent studies have indicated that the same strain of bacteria cultured in the medium with different pH values can produce divergent BEVs in terms of their contents and functional consequences (Cao et al. 2020; Müller et al. 2021; Zaghloul et al. 2023). Our study reported here clearly demonstrates that BEVs produced by LGG grown in the pH8-medium (BEV^pH8^) can stimulate the expression of FPR1/2 in macrophages to the greatest degree, leading to the strongest capacity of phagocytosis and intracellular bactericidal, compared with BEV^pH5^ and BEV^pH6.5^ (Figs. 2, 3 and 7). Therefore, both the bacterial strains and growth conditions should be worthy to consider when testing probiotic-BEVs as therapeutic candidates.

With respect to the mechanism underlying BEV^pH8^-mediated enhancement of macrophage phagocytosis and bacterial clearance, it could be largely ascribed to the strong upregulation of FPR1 and FPR2 in macrophages (Fig. 7). Numerous studies have shown that, as G protein-coupled receptors (GPCRs), FPR1 and FPR2 can trigger intracellular signaling cascades and stimulate pathways leading to increased production of ROS through the NADPH oxidase complex (Fig. 9). While excessive levels of ROS can cause oxidative stress, the clearance of bacteria in macrophages relies on the tightly regulated production of ROS. It has been reported that insufficient ROS produced by macrophages can impair phagocytosis and microbial killing (Nguyen et al. 2017). Thus, the moderate and controlled levels of ROS are crucial for maintaining immune homeostasis and effectively combating microbial infections. In our study, we observed that ROS levels were moderate but significantly increased by 1.14-fold in BEV^pH5^-treated BMDMs, 1.26-fold in BEV^pH6.5^-treated cells, and 1.56-fold in BEV^pH8^-treated cells, compared to control cells. Importantly, when FPR1/2 were inhibited by Boc2, such an increase of ROS triggered by LGG-BEVs was offset, leading to a substantial reduction in BEV-mediated bacterial clearance in macrophages (Fig. 8 and Supplementary Fig. S5A/B). These finding suggest that the FPR1/2-ROS signaling cascade is involved in the BEV-induced augmentation of bacterial killing (Fig. 9). On the other hand, the increase in FPR1/FPR2 is well known to activate PI3K-Akt pathway, which can also result in enhanced phagocytosis and microbial killing (Filina et al. 2022; Yi et al. 2024). Most importantly, Sun et al. (2023) recently reported that activation of Akt can promote the expression of MARCO, a well-characterized macrophage receptor that is responsible for the uptake of bacteria. Indeed, our results demonstrated that the expression of MARCO was remarkably increased in BEV-treated macrophages, simultaneously accompanied by dramatically elevation of FPR1/2 (Fig. 7). Consistently, the levels of phosphorylated Akt were significantly increased in these BEV-treated macrophages (Fig. 7), indicating that the PI3K-Akt pathway is activated by LGG-derived BEVs. Together, we propose here that BEV^LGG^ can upregulate the expression of FPR1/2, leading to moderately increased production of ROS and activation of the PI3K-Akt-MARCO pathway in macrophages, thereby enhancing phagocytosis and intracellular bactericidal activity (Fig. 9). Nonetheless, other contents such as LTA (albeit its level is very low), short non-coding RNAs, and short-chain fatty acids that are included in BEVs may exert possible effects on stimulating macrophage phagocytosis. Future studies would be warranted to delve deeper into the specific molecular factors involved in the interaction between BEV^LGG^ and macrophages to clarify the exact underlying mechanism.

Regarding FIP-induced sepsis model used in the present study, it would be argued why not employ traditional CLP-induced sepsis model for the in vivo study. Currently, there is a concern that CLP model exhibits significant variability due to differences in surgical techniques and environmental factors (cecal content, etc.). These inconsistencies result in a wide range of outcomes, making it difficult to standardize the model (Lewis et al. 2016). However, the FIP model has the following several advantages: Firstly, the model incorporates a diverse range of microorganisms by using feces as a source of pathogens, which mimics the clinical scenario of polymicrobial sepsis more accurately than single-pathogen models. Secondly, peritonitis can be easily induced in mice without the need for anesthesia or surgical procedures. Lastly, the FIP model provides a consistent source of bacterial load, which leads to uniformly induction of sepsis. This consistency helps minimize variations in the severity of the disease among experimental mice (Fang et al. 2020). Hence, the FIP model is considered a more optimized in vivo sepsis model due to its ease of execution and standardization. Nonetheless, one major limitation of the present study is that the mortality rate of FIP-sepsis model with injection of feces at 1.5 mg/g body weight appears a little bit higher in the vehicle-control group. To test therapeutic effects, it is strongly recommended to utilize a mouse FIP-sepsis model with ≈ 50% mortality rate in the vehicle group. Therefore, to better develop therapeutics, future studies will be needed to optimize the feces dosage for inducing sepsis with ≈ 50% mortality rate at 48–72 h post-FIP.

## Conclusion

In summary, our results indicate that BEVs produced by probiotic LGG enhance macrophage phagocytosis and bacterial clearance through the activation of FPR1/2-mediated signaling pathway, especially when BEVs are produced in culture medium under alkaline conditions. This study highlights the potential therapeutic implications of probiotics LGG-derived BEVs in treating sepsis and improving survival outcomes.

## Supplementary Information

Supplementary Material 1

Supplementary Material 2

## Data Availability

The datasets used and/or analyzed in the current study are available from the corresponding author on reasonable request. RNA-sequencing data that support the findings of this study have been deposited in the NCBI Gene Expression Omnibus (GEO) and are accessible through GEO Series accession number GSE274800.

## Abbreviations

BEVs: Bacterial extracellular vesicles
LGG: *Lactobacillus rhamnosus* Gorbach-Goldin
LP WCFS1: *Lactiplantibacillus plantarum* WCFS1
EcN: *Escherichia coli* Nissle 1917
FIP: Feces-injection-in-peritoneum
ALT: Alanine aminotransferase
AST: Aspartate aminotransferase
Cr: Creatinine
LDH: Lactate dehydrogenase
PPRs: Pathogen recognition receptors
TLRs: Toll-like receptors
CLRs: C-type lectin receptors
PAMPs: Pathogen-associated molecular patterns
ROS: Reactive oxygen species
EVs: Extracellular vesicles
ATCC: American type culture collection
MRS: De Man, rogosa and sharpe
LB: Luria-Bertani
PBS: Phosphate-buffered saline
NTA: Nanoparticle tracking analysis
DLS: Dynamic light scattering
LTA: Lipoteichoic acid
DMEM: Dulbecco’s modified eagle medium
FBS: Fetal bovine serum
BMDMs: Bone marrow-derived macrophages
Boc2: N-tert-butyloxycarbonyl-Phe-Leu-Phe-Leu-Phe
BSA: Bovine serum albumin
CFU: Colony-forming unit
MOI: Multiplicity of infection
NIH: National institutes of health
MSS: Murine sepsis score
WT: Wild-type
R.O.: Retro-orbital
PLF: Peritoneal lavage fluid
H&E: Hematoxylin and eosin
IL-6: Interleukin-6
TNF-α: Tumor necrosis factor-α
IL-1β: Interleukin-1β
MCP-1: Monocyte chemoattractant protein-1
GSEA: Gene set enrichment analysis
GAPDH: Glyceraldehyde-3-phosphate dehydrogenase
RNA-seq: RNA sequencing
TBST: Tris-buffered saline with tween 20
SEM: Standard error of the mean
ANOVA: Analysis of variance
CLP: Cecal ligation and puncture
FPRs: Formyl peptide receptors
MARCO: Macrophage receptor with collagenous structure
NADPH: Nicotinamide adenine dinucleotide phosphate
GPCRs: G Protein-coupled receptors
