# Supplementary material for: Probiotic bacteria-released extracellular vesicles enhance macrophage phagocytosis in polymicrobial sepsis by activating the FPR1/2 pathway
Source: Mol Med. 2024 Nov 14;30:216. doi: 10.1186/s10020-024-00959-9 (PMC11566284; doi:10.1186/s10020-024-00959-9)
Supplement: Supplementary file 1 — Supplementary Material 1 [file 10020_2024_959_MOESM1_ESM.docx]

**Supplementary Table S1:** Primer sequences for RT-qPCR

| **Gene** | **Forward primer (5´-3´)** | **Reverse primer (5´-3´)** |
| --- | --- | --- |
| 16S rRNA | CTTAGGGAGCGTTGAGACAGG | TAACCCAACATCTCACGACACG |
| IL-1β | GCAACTGTTCCTGAACTCAACT | ATCTTTTGGGGTCCGTCAACT |
| IL-6 | GAGGATACCACTCCCAACAGACC | AAGTGCATCATCGTTGTTCATACA |
| TNF-α | AAACCACCAAGTGGAGGAGC | ACAAGGTACAACCCATCGGC |
| MCP-1 | TTAAAAACCTGGATCGGAACCAA | GCATTAGCTTCAGATTTACGGGT |
| FPR1 | CAGGCAGGGCCTGATCAAAT | ACCGTTCACGGACTTGGATT |
| FPR2 | CACCACAGGAACCGAAGAGT | TCACTAGTCCATTGCCCAGC |
| MARCO | GACAAGCCCTTCTTCTCGCT | GGTTGTTGAACTGCTGACGG |
| GAPDH | TGCACCACCAACTGCTTAGC | GGCATGGACTGTGGTCATGAG |

RT-qPCR: Real-time polymerase chain reaction
