# Supplementary material for: Probiotic bacteria-released extracellular vesicles enhance macrophage phagocytosis in polymicrobial sepsis by activating the FPR1/2 pathway
Source: Mol Med. 2024 Nov 14;30:216. doi: 10.1186/s10020-024-00959-9 (PMC11566284; doi:10.1186/s10020-024-00959-9)
Supplement: Supplementary file 2 — Supplementary Material 2 [file 10020_2024_959_MOESM2_ESM.docx]

**Supplementary Figure S1-5**

**Supplementary Figure S1**

**
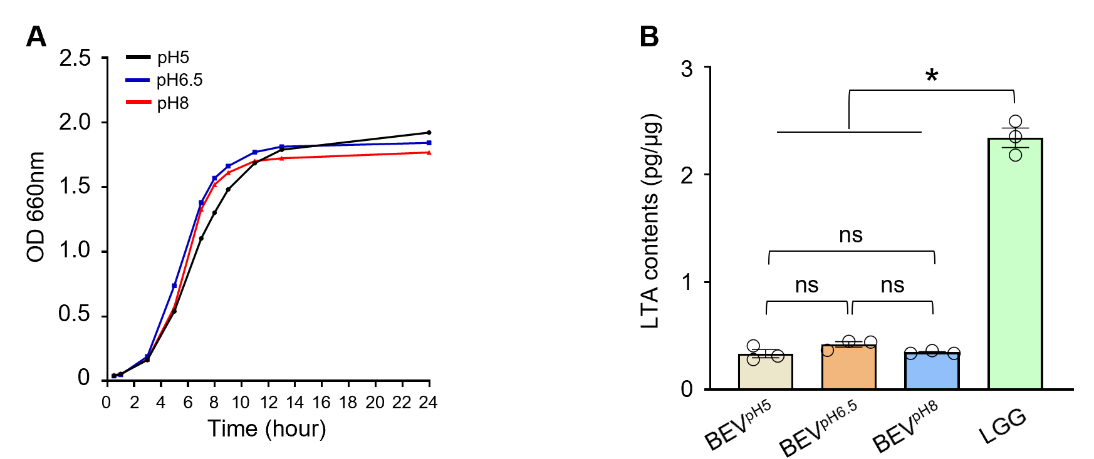
**

**
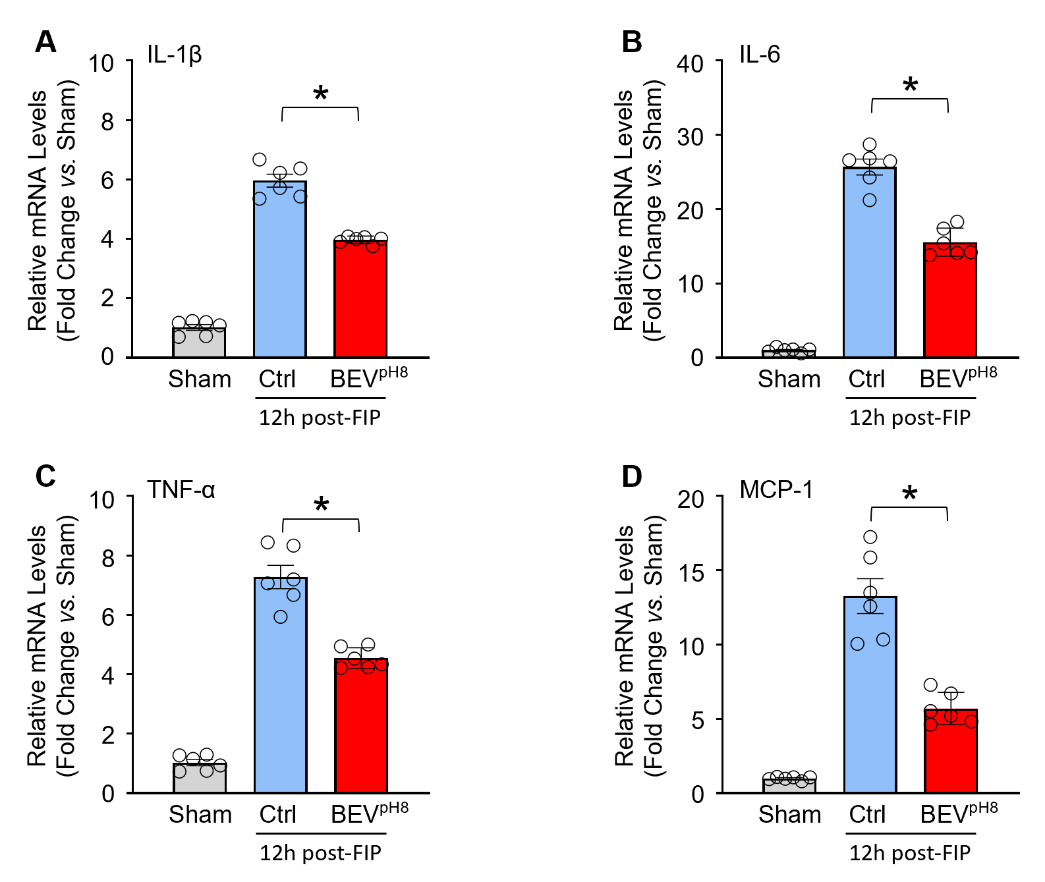
**

**Figure S2:** Relative mRNA levels of inflammatory cytokines and chemokines in lung tissues [**(A):** IL-1β, **(B):** IL-6, **(C):** TNF-α, **(D):** MCP-1] of Sham, Ctrl- and BEV^pH8^-treated mice at 12 h post-FIP by RT-qPCR (n = 6). All results are presented as mean ± SEM and analyzed by one-way ANOVA (**p* < 0.001).

**Supplementary Figure S2**

**Figure S1:** **(A)** Growth curves of LGG cultured in acidic, neutral, and alkaline medium. **(B)** LTA contents in BEVs released by LGG cultured under acidic, neutral, and alkaline conditions. All results are presented as mean ± SEM and analyzed by one-way ANOVA (**p* < 0.001, ns: non-significance).

**
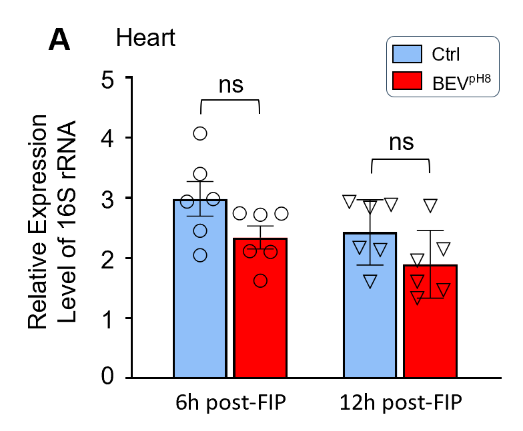
**

**Supplementary Figure S3**

**Figure S3:** Relative expression level of bacterial 16S rRNA in the heart collected from control- and BEV^pH8^-treated mice at 6 h and 12 h post-FIP by RT-qPCR (n = 6). All results are presented as mean ± SEM and analyzed by 2-way ANOVA (ns: non-significance).

**Supplementary Figure S4**

Raw264.7 macrophages BMDMs


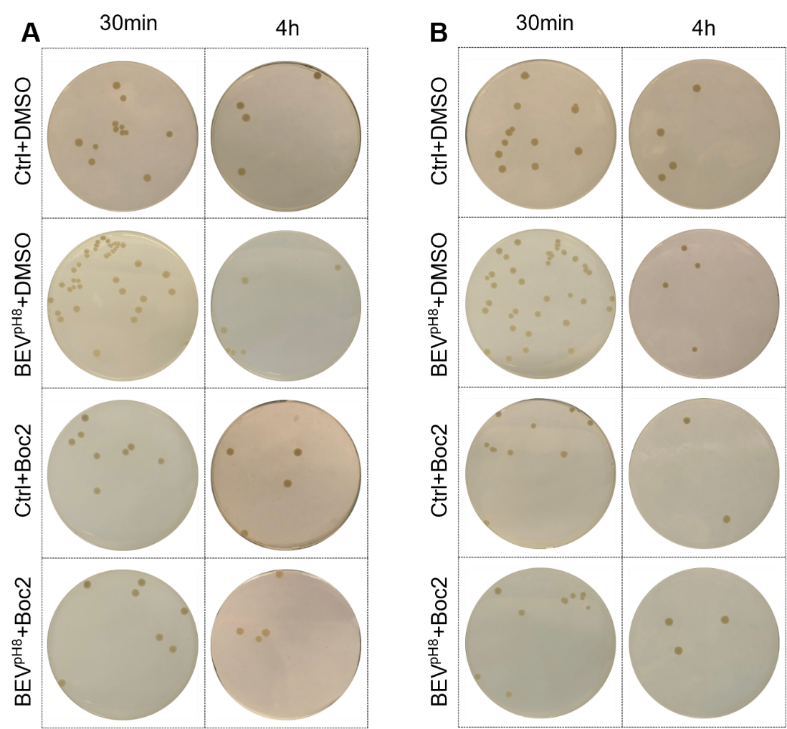


**Figure S4:** Representative images of gentamicin protection assay using live *E. coli* to detect phagocytic and bactericidal activities in Raw264.7 macrophages **(A)** and BMDMs **(B)**. Gentamicin protection assay was used to detect the phagocytic and bactericidal activities upon pre-treatment of macrophages (Raw264.7 and BMDMs) with Boc2, a specific antagonist of FPR1/2, for 2 h, followed by incubation with BEV^pH8^ for 12 h. Gentamicin (100µg/ml) was added to the cell culture medium 1 h after infection with live *E. coli* (MOI=20). After 30 min, cell lysates were extracted with serial dilution, then plated on LB agar plates. The CFUs were measured as an indicator for phagocytosis capacity of Raw264.7 macrophages **(A)** and BMDMs **(B)**. Then, CFUs were determined in lysates of Raw264.7 macrophages and BMDMs at 4 h after the addition of gentamicin to assess the number of bacteria remained within macrophages. The killing percentages of macrophages were calculated as described in the section of Materials & Methods.

**Supplementary Figure S5**


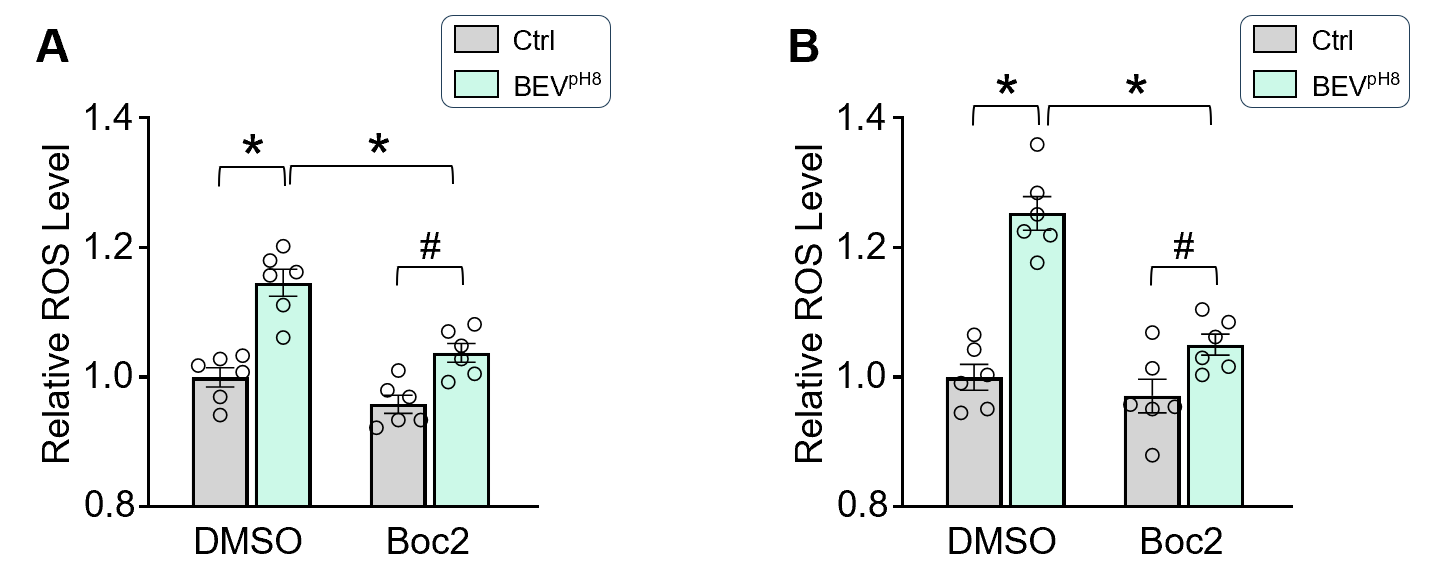


**Figure S5:** Raw264.7 macrophages **(A)** and BMDMs **(B)** were treated with BEV^pH8^ in the presence or absence of Boc2 for 12 h, followed by incubation with heat-inactivated *E. coli* for 30 min, and then ROS levels were assayed. All results are presented as mean ± SEM and analyzed by 2-way ANOVA (**p* < 0.001, ^#^*p* < 0.05).
